# Functional connectome fingerprint of holistic–analytic cultural style

**DOI:** 10.1093/scan/nsab080

**Published:** 2021-06-23

**Authors:** Siyang Luo, Yiyi Zhu, Shihui Han

**Affiliations:** Department of Psychology, Guangdong Key Laboratory of Social Cognitive Neuroscience and Mental Health, Guangdong Provincial Key Laboratory of Brain Function and Disease, Sun Yat-sen University, Guangzhou 510006, China; Department of Psychology, Guangdong Key Laboratory of Social Cognitive Neuroscience and Mental Health, Guangdong Provincial Key Laboratory of Brain Function and Disease, Sun Yat-sen University, Guangzhou 510006, China; School of Behavioral and Brain Sciences, The University of Texas at Dallas, Richardson, TX 75080, USA; School of Psychological and Cognitive Sciences, PKU-IDG/McGovern Institute for Brain Research, Beijing Key Laboratory of Behavior and Mental Health, Peking University, Beijing 100080, China

**Keywords:** connectome, culture, holistic–analytic thinking

## Abstract

Although research in the field of cultural psychology and cultural neuroscience has revealed that culture is an important factor related to the human behaviors and neural activities in various tasks, it remains unclear how different brain regions organize together to construct a topological network for the representation of individual’s cultural tendency. In this study, we examined the hypothesis that resting-state brain network properties can reflect individual’s cultural background or tendency. By combining the methods of resting-state magnetic resonance imaging and graph theoretical analysis, significant cultural differences between participants from Eastern and Western cultures were found in the degree and global efficiency of regions mainly within the default mode network and subcortical network. Furthermore, the holistic–analytic thinking style, as a cultural value, provided a partial explanation for the cultural differences on various nodal metrics. Validation analyses further confirmed that these network properties effectively predicted the tendency of holistic–analytic cultural style within a group (*r* = 0.23) and accurately classified cultural groups (65%). The current study establishes a neural connectome representation of holistic–analytic cultural style including the topological brain network properties of regions in the default mode network, the basal ganglia and amygdala, which enable accurate cultural group membership classification.

## Introduction

Research in cultural neuroscience has revealed that cultural behaviors or values are closely associated with neural activities (Rule *et al.*, 2013; Han and Ma, 2014; Sasaki and Kim, 2017). Engaging in tasks related to a culture repeatedly will create culturally patterned neural activities and will be reflected by the anatomical or functional characteristics of the brain (Kitayama and Tompson, 2010; Kitayama and Uskul, 2011). In particular, Kitayama *et al.* (2017) discovered that among Japanese individuals, the brain volume of the orbitofrontal cortex is negatively correlated with interdependence, which is an important cultural value among East Asians (Markus and Kitayama, 1991). Another study also found that independence is associated with an increase in gray-matter volume in regions such as the ventromedial prefrontal cortex (vmPFC), right dorsolateral prefrontal cortex (DLPFC) and right rostrolateral prefrontal cortex (RLPFC) among Chinese individuals (Wang *et al.*, 2017). These studies indicate that typical cultural values are interrelated with the structural features of the brain, and the neural mechanism of cultural expression may involve different brain regions.

In recent decades, using functional magnetic resonance imaging (fMRI) methods, researchers have also explored how culture affects the functional properties of the brain. During different culturally related tasks, different brain activation patterns have been found across cultural groups. For example, Zhu *et al.* (2007) discovered that the overlapping neural correlates of reflection on oneself and others close to the self are specific to Chinese individuals and are not shared by Westerners. Research has also revealed that in self-judgment tasks, Caucasian Americans exhibit higher levels of activation in the bilateral thalamus, right putamen (PUT), bilateral cuneus, right insula, bilateral cerebellum and right superior frontal gyrus, whereas native Japanese individuals show greater activation in the left middle temporal gyrus (Chiao *et al.*, 2009). Likewise, self-judgment stimulates greater neural medial prefrontal cortex ( activity in the medial prefrontal cortex (mPFC) among Danish participants but greater neural activity in the temporoparietal junction (TPJ) among Chinese participants (Ma *et al.*, 2014). In addition, regarding the aspect of emotion recognition, emerging evidence also highlights the crucial role of culture in modulating the amygdala (AMYG) response. For example, greater AMYG activation was found in processing fear expressed by members of the same cultural group (Chiao *et al.*, 2008). Other studies also revealed that Asian participants showed a stronger AMYG response to Caucasian emotional faces than European participants did, and a longer duration of stay in a foreign culture is associated with a lower level of AMYG activation among Asians (Derntl *et al.*, 2009, 2012). Additionally, in object or emotion-processing tasks, significant cultural differences were found in the prefrontal cortex, TPJ and subcortical areas (e.g. ventral striatal; Gutchess *et al.*, 2006; de Greck *et al.*, 2012; Park *et al.*, 2016, 2018). A quantitative meta-analysis (Han and Ma, 2014) summarized that in social or nonsocial processes, distinct neural network activities are shown in East Asians and Westerners. Activity exhibited by the key regions in the social brain network, such as the mPFC, TPJ, anterior cingulate cortex (ACC) and anterior insula, are related to culture. The study also found that in social cognitive or affective tasks, East Asians show increased brain activity in the regions associated with the functions of theory of mind, self-perception and self-control/emotion regulation, while Westerners show enhanced activities in the regions that are important for the functions of self-reflection, socioemotional processing and emotion/empathy responses (Han and Ma, 2014).

The abovementioned findings suggest that culture modulates the functional properties of the brain, and the involvement of different brain regions suggests that the representation of culture may depend on the brain network. Noticeably, regions that have been revealed to have cultural differences are mainly distributed in subcortical areas (e.g. AMYG and ventral striatal) and in the default mode network (DMN; e.g. mPFC, TPJ and ACC; Andrews-Hanna *et al.*, 2010). Additionally, previous findings were obtained mostly by comparing brain activation across cultural groups during specific tasks; whether people from different cultures have distinct brain network properties or culturally patterned functional properties in a general state remains unknown. In this study, we address this gap by comparing spontaneous brain activity measured by the resting-fMRI (R-fMRI) method. Compared to task-based fMRI, which illustrates the function of different brain regions during specific tasks (Shimony *et al.*, 2009), R-fMRI enables us to examine the functional connections between different regions in a general state (Shen *et al.*, 2010) and helps identify the functional architecture and the resting networks of the brain (Lee *et al.*, 2013). Moreover, research has shown that R-fMRI signals can effectively predict people’s psychological tendencies and can be used to clarify different types of neurologic and psychiatric diseases (Chen *et al.*, 2011; Pizoli *et al.*, 2011; Baur *et al.*, 2013; Lee *et al.*, 2013).

Further research using R-fMRI also proposed the concept of the human connectome, which emphasizes the importance of viewing the brain as a complex, interconnected network with different regions working cooperatively (Sporns *et al.*, 2005; van den Heuvel and Hulshoff Pol, 2010; Sporns, 2013). To unravel the human connectome, rational informative mapping of the brain is equally important (Fornito *et al.*, 2013). It has been shown that graph theory, which abstracts the brain as a graph with different brain regions as nodes and their interrelationships as edges, is appropriate for analyzing brain connectivity data (i.e. correlation data collected with R-fMRI; He and Evans, 2010; Braun *et al.*, 2012; Fornito *et al.*, 2013) and is an effective way to understand topological brain network characteristics (Bullmore and Sporns, 2009). Graph theoretical analysis also provides different metrics for measuring the nodal functional properties in the whole-brain network, which extends the understanding of regional activation measured by task-based fMRI.

In the current study, we investigated the cultural representation in functional brain networks through R-fMRI and graph theoretical analysis. We first examined whether people from different cultural backgrounds (namely, Westerners *vs* Chinese individuals) exhibit diverse functional properties in different brain regions. For people from Western cultural backgrounds, who are more likely to endorse an independent construal of self and emphasize the internal attributes of self (Markus and Kitayama, 1991), brain regions that are involved in self-related processing may be relatively important in the brain network. Thus, we hypothesized that regions in the DMN (e.g. mPFC), which have been revealed as crucial areas for maintaining independent self-construal (Han *et al.*, 2008; Kitayama and Park, 2010; Kitayama *et al.*, 2017; Wang *et al.*, 2017) and self-consciousness (Qin and Northoff, 2011; Davey *et al.*, 2016), would be crucial nodes in Westerners’ brain networks. In contrast, for East Asians, who are more interdependent and underline harmonious relationships with others (Markus and Kitayama, 1991), brain regions that contribute to social information processing may be more critical in their brain network. As emotion is an essential aspect of social information that influences social interaction (Van Kleef, 2009), previous work provides abundant evidence that culture affects emotional processing. For instance, past research has suggested that East Asians or people who are more interdependent are more sensitive to emotional information (Ishii *et al.*, 2011; Ma-Kellams and Blascovich, 2012). Moreover, Kitayama *et al.* (2006) found that Japanese people experience more socially engaging emotions (e.g. friendly feelings, respect and shame), whereas Americans experience more socially disengaging emotions (e.g. superiority, anger and frustration). Tsai *et al.* (2006) discovered that European Americans place a higher value on high-arousal positive emotions, while Chinese individuals are more disposed to experiencing low-arousal positive emotions. Combined with studies that have revealed that the AMYG and subcortical areas (e.g. basal ganglia) are pivotal in processing social information and emotions (LeDoux, 1992; Johnson, 2005; Bhanji and Delgado, 2014; McFadyen *et al.*, 2020) and that the activation of the AMYG and subcortical areas is modulated by culture (Gutchess *et al.*, 2006; Chiao *et al.*, 2008; de Greck *et al.*, 2012; Park *et al.*, 2016, 2018), we hypothesized that the AMYG and subcortical areas would be important nodes in the brain networks of Chinese individuals.

Furthermore, we explored whether cultural differences in brain network properties were interrelated with a typical culture value. We focused on the holistic–analytic thinking style, which is a crucial cultural value that affects an individual’s cognitive processes (e.g. attention) and social interaction. Abundant evidence shows that people from East Asian cultures (e.g. China, Korea and Japan) tend to exhibit holism in perception and cognition representation, which is characterized by paying attention to both a focal object and the background and comprehending behavior and causality according to the relationships between specific elements, whereas people from Western culture are more likely to process information in a more analytical way, characterized by paying more attention to a focal object and understanding behavior and causality according to established rules or attributes rather than corresponding contexts (Morris and Peng, 1994; Choi *et al.*, 1999, 2003; Nisbett and Miyamoto, 2005). Researchers suggest that cultural differences in holistic–analytic thinking styles are rooted in how social relations are organized in different cultures. In interdependent social environments, individuals may encounter various situations in which social relationships should be considered, behave or make attributions according to different contexts and reconcile inconsistencies to promote social harmony (Cheon *et al.*, 2018). Through long-term cultural practices, a holistic thinking style will be internalized and reinforced in an interdependent culture (Kitayama *et al.*, 2009). In contrast, independent social environments may promote the development of analytic thinking. Based on research that has revealed that psychological activities associated with analytic thinking, such as rumination (Watkins and Teasdale, 2001; Rimes and Watkins, 2005; Moulds *et al.*, 2007) and focal object processing (compared to background processing; Kitayama *et al.*, 2003; Nisbett and Masuda, 2003; Senzaki *et al.*, 2014), involve different regions in the DMN (Gutchess *et al.*, 2006; Hedden *et al.*, 2008; Berman *et al.*, 2011; Zhou *et al.*, 2020), we hypothesized that the importance of DMN nodes in the brain network (measured by graph metrics) would be associated with analytic thinking. Compared to analytic thinking, holistic thinking increases attention to the relationship between a focal object and its contexts (Nisbett and Miyamoto, 2005). Combining cross-cultural studies that showed that holistic thinkers are more proficient in integrating contradictory emotional signals (Masuda *et al.*, 2008; Goto *et al.*, 2010; Ishii *et al.*, 2010) and neuroscience studies that revealed that AMYG integrates emotional expression cues (Cristinzio *et al.*, 2010; Sato *et al.*, 2010) and that subcortical areas (e.g. basal ganglia) are crucial areas for attention switching (Casey *et al.*, 2004; Van Schouwenburg *et al.*, 2010, 2014), we hypothesized that holistic thinking would emphasize the role of AMYG and subcortical areas. Finally, we explored the feasibility of using functional metrics as a cultural fingerprint to predict the tendency toward a holistic–analytic thinking style and to classify individuals into cultural groups.

## Study 1

Study 1 sought to examine cultural differences in graph metrics across the functional brain network. Based on R-fMRI data, a functional brain network was constructed for each participant, and graph metrics were calculated accordingly. Through comparison of graph metrics across two cultural groups, we found that East Asians (i.e. Chinese individuals) and Westerners have distinct brain networks.

### Method

#### Participants.

Study 1 gathered R-fMRI data from both Chinese and Western samples. The Chinese sample contained 306 healthy adults (91 females; age = 21.05 ± 2.43 years). R-fMRI data of the Western sample were adopted from multicenter datasets and included 315 healthy adults (168 females; age = 31.93 ± 15.61 years). The Western sample was selected from the 1000 Functional Connectomes Project dataset (FCP, www.nitrc.org/projects/fcon_1000/, Biswal *et al.*, 2010). We selected datasets that match the TR and magnet field strengths of the Chinese sample. Demographic details and scanning parameters of each dataset are provided in Table 1.

**Table 1.** Demographic details and scan parameters of each dataset from the FCP

		*N*	Sex	Age	TR	Slices	Timepoints
AnnArbor_a	3 T	25	22 M/3 F	13–40	2	40	295
Bangor	3 T	20	20 M/0 F	19–38	2	34	265
Dallas	3 T	24	12 M/12 F	20–71	2	36	115
ICBM	3 T	86	41 M/45 F	19–85	2	23	128
Newark	3 T	19	9 M/10 F	21–39	2	32	135
NewYork_a	3 T	84	43 M/41 F	7–49	2	39	192
NewYork_b	3 T	20	8 M/12 F	18–46	2	33	175
Oxford	3 T	22	12 M/10 F	20–35	2	34	175
PaloAlto	3 T	17	2 M/15 F	22–46	2	29	235

#### Image acquisition.

Scanning parameters for data from the FCP are presented in Table 1. The Chinese data were acquired using a 3.0 T scanner with a standard head coil. Functional images were acquired using T2-weighted, gradient-echo and echo-planar imaging (EPI) sequences sensitive to BOLD signals (64 × 64 × 32 matrix with 3.75 × 3.75 × 5 mm^3^ spatial resolution, repetition time = 2000 ms, echo time = 30 ms, flip angle = 90° and field of view = 24 × 24 cm). A high-resolution T1-weighted structural image (512 × 512 × 180 matrix with a spatial resolution of 0.47 × 0.47 × 1.0 mm^3^, repetition time = 8.204 ms, echo time = 3.22 ms and flip angle = 12°) was acquired before the functional scans. Participants underwent a 5 min scan and were instructed to relax and keep their eyes closed but to not fall asleep during scanning.

#### Data preprocessing.

All functional imaging data were preprocessed using Statistical Parametric Mapping 8 ( http://www.fil.ion.ucl.ac.uk/spm/) and the Data Processing Assistant for Resting-State fMRI (Yan and Zang, 2010). Before data processing, we checked the quality of the R-fMRI data obtained from the FCP, and 43 participants with low-quality raw images were excluded. The first five volumes of each participant’s resting-state data (except for the Dallas dataset) were discarded to reduce the effect of unstable BOLD signals at the beginning of scanning. Image data of each participant were truncated to 115 timepoints to coordinate with the data with the fewest timepoints among the datasets. The remaining fMRI data were slice acquisition corrected according to the number of slices and the slice order of each dataset. Head motion correction was conducted thereafter using head motion criteria of 3° and 3 mm. Six Chinese participants and three Westerners were excluded due to excessive head motion. fMRI data were then spatially normalized to the standard EPI template in Montreal Neurological Institute (MNI) space with a resampled resolution of 3 × 3 × 3 mm^3^ and spatially smoothed using a 4 mm Gaussian kernel. Normalization was conducted based on EPI images because several studies have suggested that this approach increases registration accuracies and is more accurate than normalization based on T1-weighted images (Calhoun *et al.*, 2017; Dohmatob *et al.*, 2018; Faghiri *et al.*, 2018; Weis *et al.*, 2020). Detrending and temporal filtering (0.01–0.08 Hz) were then applied to remove low-frequency drift and high-frequency physiological noise. Finally, 24 head motion parameters (Friston *et al.*, 1996) and two potential nuisance signals, including cerebrospinal fluid and white matter, were regressed out from time courses at each voxel. Given that studies have suggested that global signal regression is a controversial step that may induce artifactual negative correlations (Fox *et al.*, 2009; Murphy *et al.*, 2009; Weissenbacher *et al.*, 2009; Anderson *et al.*, 2011; Saad *et al.*, 2012; Nalci *et al.*, 2017) and that global signals include significant neural correlates (Schölvinck *et al.*, 2010; Wong *et al.*, 2013; Wen and Liu, 2016) and may provide meaningful neurobiological information (Yang *et al.*, 2014), we did not regress the global signal out. The preprocessing decreased the sample to 269 Westerners and 300 Chinese participants for the following analyses.

#### Graph theoretical analysis

##### Network construction.

To define the nodes of the network for further processing, we employed the Automated Anatomical Labeling atlas (Tzourio-Mazoyer *et al.*, 2002) to segment each participant’s images into 90 cortical and subcortical regions of interest. To estimate the network edges, we first calculated Pearson correlation coefficients between the regional mean time series of all possible pairs of nodes, resulting in a 90 × 90 correlation matrix for each participant. In our subsequent analysis, individual correlation matrices were converted into binary matrices A*_ij_* = [a*_ij_*] using a predefined threshold. In binary matrices A*_ij_*, a*_ij_* was set to 1 if the value of the correlation between regions *i* and *j* was larger than the threshold and 0 otherwise. Before this process, we set the negative correlations to zero and retained the positive correlations because negative correlations may lead to low test–retest reliability (Wang *et al.*, 2011).

##### Network analysis.

Instead of selecting a single threshold for correlation analyses, we applied sparsity thresholds *S* to all correlation matrices. *S* was computed as the ratio of the number of existing edges divided by the maximum possible number of edges in a network (Bullmore and Bassett, 2011). This approach was performed by applying a specific correlation coefficient threshold for each participant. Therefore, all resulting matrices were normalized to have the same number of edges, which minimized the effects of possible discrepancies in the overall correlation strength between groups (Achard and Bullmore, 2007; He *et al.*, 2009). We applied a threshold range of 0.10 ≤ *S* ≤ 0.30 to each binary matrix, which was similar to previous research (Khundrakpam *et al.*, 2017). Nodal network topological properties were calculated under each threshold. Moreover, we calculated the area under the curve (AUC) for each metric over the sparsity threshold range to provide a summary of the topological characterization of the brain network. To determine the association between culture and the importance of different brain regions, we focused on metrics concerning each node’s effect on the overall brain network. These metrics included the degree *K_i_* and the global efficiency *E_i_global_*.

The degree of each node, *K_i_*, is defined as the number of direct connections between node *i* and other nodes in the network and thus reflects the number of direct neighbors of node *i* (Bullmore and Sporns, 2009). The nodal global efficiency, *E_i_global_*, is the inverse of the harmonic mean of the length between node *i* and all other nodes in the network.

$${E_{i\_global}} = {1 \over {N - 1}}\mathop \sum \limits_{\substack{
{j \in G} \cr
{j \ne i}} } {1 \over {min\left\{ {{L_{i,j}}} \right\}}}$$

The Gretna toolbox (http://www.nitrc.org/projects/gretna/, Wang *et al.*, 2015) was used to calculate the functional connectivity strength matrices (r-score matrices and z-score matrices) and to conduct analyses of graph theoretical metrics. The group differences (Chinese individuals *vs* Westerners) in the AUC of the degree and global efficiency of each node were first estimated by conducting a multivariate analysis of covariance (MANCOVA) and an analysis of covariance (ANCOVA), which included head motion, sex, age and slice number as covariates. To account for multiple comparisons of the AUC of nodal metrics (90 nodes here), false discovery rate (FDR) correction was applied for MANCOVAs and ANCOVAs separately. Nodes with significant cultural effects, as revealed by MANCOCAs and ANCOVAs, were considered nodes related to cultural differences between Westerners and Chinese people.

#### Supplementary analyses.

To test the robustness of our findings, we also conducted supplementary analyses to examine the potential influences of global signals, negative correlations and different brain parcellations.

### Results

We first examined the age and sex distributions among the two cultural samples before fMRI data analysis. The distribution of age significantly differed across Westerners and Chinese participants (30.11 ± 14.60 *vs* 21.08 ± 2.45 years, *t*(281.51) = 10.02, *P* < 0.001). The sex ratio in the Western and Chinese samples also differed significantly (*χ^2^*(1569) = 28.62, *P* < 0.001). Therefore, in the MANCOVAs and ANVOVAs for assessing cultural differences in each node’s degree and global efficiency, age and sex were included as covariates.

**Fig. 1.**
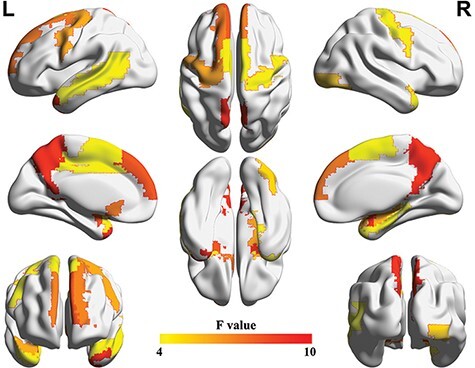
Results of the MANCOVA analyses of the AUC for the degree and global efficiency.

The MANCOVAs revealed significant group differences in the AUC of degree and global efficiency for 26 nodes (FDR corrected, *P* < 0.05; Figure 1, Table 2). These nodes included the bilateral precentral gyrus (PreCG), left dorsal superior frontal gyrus (SFGdor), bilateral supplementary motor area, left olfactory cortex (OLF), bilateral mPFC, left dorsal anterior cingulate cortex (DACC), right hippocampus (HIP), right parahippocampal gyrus (PHG), bilateral AMYG, right inferior occipital gyrus (IOG), bilateral precuneus (PCUN), left caudate (CAU), bilateral PUT, bilateral pallidum (PAL), bilateral superior temporal pole (TPOsup), left middle temporal gyrus and bilateral middle temporal pole (TPOmid) (*Fs*(2557) = 4.57–21.78, *ps* = 7.83 × 10^−10^–0.011).

**Table 2.** Results of MANCOVA and ANCOVA analyses of the AUC for the degree and global efficiency

Nodes	MANCOVA			ANCOVA of degree				ANCOVA of global efficiency			
	*F*	*P*	*η_p_^2^*	*F*	*P*	*η_p_^2^*	West/East	*F*	*P*	*η_p_^2^*	West/East
L.PreCG	6.59*	0.001	0.023	13.17*	3.10 × 10^−4^	0.023	W	11.08*	9.32 × 10^−4^	0.019	W
R.PreCG	5.55*	0.004	0.020	11.11*	9.17 × 10^−4^	0.020	W	8.95*	0.003	0.016	W
L.SFGdor	8.24*	2.98 × 10^−4^	0.029	15.10*	1.14 × 10^−4^	0.026	W	9.67*	0.002	0.017	W
L.SMA	4.65*	0.010	0.016	1.44	0.230	0.003		0.01	0.920	1.81 × 10^−5^	
R.SMA	4.57*	0.011	0.016	0.11	0.740	1.97 × 10^−4^		2.37	0.124	0.004	
L.OLF	7.32*	7.29 × 10^−4^	0.026	8.57*	0.004	0.015	E	14.49*	1.57 × 10^−4^	0.025	E
L.mPFC	8.39*	2.57 × 10^−4^	0.029	16.71*	5.00 × 10^−5^	0.029	W	13.55*	2.55 × 10^−4^	0.024	W
R.mPFC	7.22*	8.00 × 10^−4^	0.025	14.35*	1.68 × 10^−4^	0.025	W	11.37*	8.00 × 10^−4^	0.020	W
L.DACC	5.50*	0.004	0.019	3.90	0.049	0.007		7.42*	0.007	0.013	E
R.HIP	6.37*	0.002	0.022	12.38*	4.69 × 10^−4^	0.022	E	7.24*	0.007	0.013	E
L.PHG	3.87	0.021	0.014	5.16	0.023	0.009		7.67*	0.006	0.014	E
R.PHG	5.26*	0.005	0.019	8.82*	0.003	0.016	E	10.49*	0.001	0.018	E
L.AMYG	10.25*	4.27 × 10^−5^	0.035	13.52*	2.59 × 10^−4^	0.024	E	20.43*	7.54 × 10^−6^	0.035	E
R.AMYG	15.20*	3.74 × 10^−7^	0.052	26.17*	4.30 × 10^−7^	0.045	E	29.83*	7.11 × 10^−8^	0.051	E
L.IOG	3.29	0.038	0.012	5.00	0.026	0.009		6.59*	0.011	0.012	E
R.IOG	5.72*	0.003	0.020	6.76*	0.010	0.012	E	11.15*	8.98 × 10^−4^	0.020	E
L.PCUN	17.65*	3.71 × 10^−8^	0.060	34.88*	6.08 × 10^−9^	0.059	W	26.26*	4.11 × 10^−7^	0.045	W
R.PCUN	11.59*	1.17 × 10^−5^	0.040	22.63*	2.50 × 10^−6^	0.039	W	16.26*	6.29 × 10^−5^	0.028	W
L.CAU	7.73*	4.86 × 10^−4^	0.027	9.34*	0.002	0.016	W	1.12	0.291	0.002	
L.PUT	9.06*	1.34 × 10^−4^	0.032	13.91*	2.12 × 10^−4^	0.024	E	18.14*	2.41 × 10^−5^	0.031	E
R.PUT	9.85*	6.25 × 10^−5^	0.034	13.56*	2.54 × 10^−4^	0.024	E	19.65*	1.12 × 10^−5^	0.034	E
L.PAL	20.59*	2.37 × 10^−9^	0.069	39.67*	6.10 × 10^−10^	0.066	E	36.81*	2.41 × 10^−9^	0.062	E
R.PAL	21.78*	7.83 × 10^−10^	0.073	41.81*	2.20 × 10^−10^	0.070	E	38.27*	1.19 × 10^−9^	0.064	E
L.TPOsup	4.68*	0.010	0.017	3.82	0.051	0.007		7.40*	0.007	0.013	E
R.TPOsup	6.66*	0.001	0.023	9.85*	0.002	0.017	E	13.13*	3.16 × 10^−4^	0.023	E
L.MTG	4.77*	0.009	0.017	2.25	0.135	0.004		0.14	0.713	2.43 × 10^−4^	
L.TPOmid	13.66*	1.62 × 10^−6^	0.047	22.61*	2.53 × 10^−6^	0.039	E	26.85*	3.08 × 10^−7^	0.046	E
R.TPOmid	5.28*	0.005	0.019	2.05	0.153	0.004		7.66*	0.006	0.014	E

*Note*: W represents a significantly higher value among Westerners; E represents a significantly higher value among East Asians (i.e. Chinese individuals). Abbreviations: L. PreCG, left precental gyrus; R. PreCG, right precental gyrus; L. SFGdor, left dorsal superior frontal gyrus; L. SMA, left supplementary motor area; R. SMA, right supplementary motor area; L. OLF, left olfactory cortex; L. mPFC, left medial prefrontal cortex; R. mPFC, right medial prefrontal cortex; L. DACC, left dorsal anterior cingulate cortex; R. HIP, right hippocampus; L. PHG, left parahippocampal gyrus; R. PHG, right parahippocampal gyrus; L. AMYG, left amygdala; R. AMYG, right amygdala; L. IOG, left inferior occipital gyrus; R. IOG, right inferior occipital gyrus; L. PCUN, left precuneus; R. PCUN, right precuneus; L. CAU, left caudate; L. PUT, left putamen; R. PUT, right putamen; L. PAL, left pallidum; R. PAL, right pallidum; L. TPOsup, left superior temporal pole; R. TPOsup, right superior temporal pole; L. MTG, left middle temporal gyrus; L. TPOmid, left middle temporal pole; R. TPOmid, right middle temporal pole.

* *P* < 0.05, FDR corrected.

**Fig. 2.**
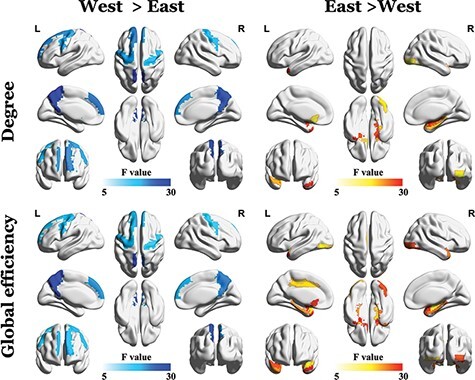
Results of ANCOVA analyses of the AUC for the degree and global efficiency. West > East represents a significantly higher value among Westerners; East > West represents a significantly higher value among East Asians (i.e. Chinese individuals).

The ANOVAs on the AUCs of degree of all nodes revealed significant group differences in the bilateral PreCG, left SFGdor, left OLF, bilateral mPFC, right HIP, right PHG, bilateral AMYG, right IOG, bilateral PCUN, left CAU, bilateral PUT, bilateral PAL, right TPOsup and left TPOmid (FDR corrected, *P* < 0.05; Figure 2, Table 2). The degrees of bilateral PreCG, left SFGdor, bilateral mPFC, bilateral PCUN and left CAU were significantly higher among Westerners (*Fs*(1558) = 9.34–34.88, *ps* = 6.08 × 10^−9^–0.002). The degrees of the left OLF, right HIP, right PHG, bilateral AMYG, right IOG, bilateral PUT, bilateral PAL, right TPOsup and left TPOmid were, however, significantly higher among Chinese participants (*Fs*(1558) = 6.76–41.81, *ps* = 2.20 × 10^−10^–0 .010).

The ANOVAs on the AUCs of global efficiency of all nodes demonstrated significant group differences in the bilateral PreCG, left SFGdor, left OLF, bilateral mPFC, left DACC, right HIP, bilateral PHG, bilateral AMYG, bilateral IOG, bilateral PCUN, bilateral PUT, bilateral PAL, bilateral TPOsup and bilateral TPOmid (FDR corrected, *P* < 0.05; Figure 2, Table 2). Westerners showed higher global efficiency in the bilateral PreCG, left SFGdor, bilateral mPFC, and bilateral PCUN (*Fs*(1558) = 8.95–26.26, *ps* = 4.11 × 10^−7^–0.003), while Chinese participants showed significantly higher global efficiency in the left OLF, left DACC, right HIP, bilateral PHG, bilateral AMYG, bilateral IOG, bilateral PUT, bilateral PAL, bilateral TPOsup and bilateral TPOmid (*Fs*(1558) = 6.59–38.27, *ps* = 1.19 × 10^−9^–0.011).

Supplementary analyses to address the potential effects of global signals, negative correlations and brain parcellations revealed similar results (see Supplementary Tables S1–S3).

To summarize, using MANCOVAs and ANCOVAs, we discovered that people from different cultural groups have distinct characteristics in the topological features of their brain networks. Nineteen nodes showed consistent cultural differences in both degree and global efficiency metrics. In general, the nodes distributed in the DMN (e.g. bilateral mPFC and PCUN) were more important in Westerners’ brain network, whereas nodes distributed in subcortical areas (e.g. AMYG and PUT) were more important for Chinese participants.

## Study 2

Although the results of Study 1 suggest group differences in the organization of functional brain networks between Chinese individuals and Westerners, it remains unclear how graph metrics and the relationships between nodes are associated with culturally specific cognitive styles such as the holistic–analytic thinking style. Therefore, based on the 19 nodes identified in Study 1, Study 2 further examined whether these nodes’ degree, global efficiency and their respective functional connectivity with the other nodes were associated with a holistic–analytic thinking style. Finally, we conducted intrasample and cross-sample validation analyses to investigate the efficiency of using culturally related brain network properties to predict individuals’ holistic–analytic thinking style and cultural background.

### Method

#### Participants.

Data from the Chinese sample in Study 1 were used.

#### Measure.

Participants completed the Analysis-Holism Scale (AHS) to measure analytic versus holistic thinking tendencies (Choi *et al.*, 2007) after scanning. The scale measures four factors involved in analytic–holistic thinking style: causality, attitude toward contradictions, perception of change and locus of attention. A higher composite score indicates a stronger inclination toward the holistic thinking style, whereas a lower composite score indicates a stronger inclination toward the analytic thinking style.

#### Data preprocessing and network analysis.

The procedures for data preprocessing and network metric analyses were similar to those in Study 1. Because each Chinese participant was scanned for 5 min (150 timepoints), after excluding the first 5 timepoints, 145 timepoints were used for data preprocessing and network analysis.

#### Data analyses

##### Partial correlation analyses.

In the first step, we investigated the relationship between the AUCs of the degree and global efficiency of the 19 nodes (identified in Study 1) and the AHS score. We conducted partial correlation analyses, controlling for the effects of sex, age and head motions. For the nodes that had at least one network metric significantly correlated with the AHS score, we further calculated the functional connectivity between the nodes and the other 89 nodes in the network separately. The functional connectivity was calculated through the correlation of the time series between node pairs. A higher score indicates a higher functional connectivity between two nodes. Additionally, we conducted partial correlation analyses to explore the relationship between the functional connectivity of these nodes and the AHS score.

##### K-fold cross-validation.

In the procedures above, the AUC of the degree of seven nodes and the AUC of the global efficiency of three nodes were significantly correlated with the AHS score. Thus, these network metrics were used to establish cultural representation models for predicting individuals’ holistic thinking style (Study 2 sample; intrasample analysis) and cultural group (Study 1 sample; cross-sample analysis). For the intrasample analysis, we tested whether the metrics could be used to predict the AHS score in the Chinese sample, and for the cross-sample analysis, we explored whether these metrics could be used to classify Westerners and Chinese individuals using the data from Study 1.

##### Intrasample validation analysis.

Before conducting the intrasample validation analysis, we unified the correlation directions between the AHS score and the nodal degree as well as the global efficiency. The unified metrics were averaged to two variables for each participant whose data was included in Study 2. T_deg_ represents the mean of the degree of seven nodes, and T_eg_ represents the mean of the global efficiency of three nodes. K-fold cross-validation (K = 10) was conducted to examine the predictive power of T_deg_ and T_eg_ in predicting the AHS score. In 10-fold cross-validation, data were first randomly separated into 10-folds and then repeated 10 times to use 9-folds for training (i.e. training set) and a left fold for validation (i.e. test set). The training set was used to fit a linear regression model between the AHS score and the two combined variables (i.e. T_deg_ and T_eg_). Subsequently, the linear regression model was applied to the test set to generate predicted AHS scores. After this iterative process, the predictive power was assessed by calculating the correlation between the predicted and observed AHS scores. The statistical significance was also estimated by comparison to the null distribution generated by the permutation test. In the permutation test, we randomly assigned the AHS scores to different participants and repeated the 10-fold cross-validation procedures 1000 times to generate a null distribution of the predictive power. We evaluated that whether the observed predictive power was significantly higher than the 95% confidence interval (CI; generated by recording the values in the 25th and 975th percentile positions) of the null distribution.

##### Cross-sample validation analysis.

In the cross-sample validation analysis, we first extracted the 10 metrics in the data from Study 1 for Westerners and Chinese participants, and we combined the 10 metrics into two variables representing the mean of the degree of seven nodes (T_deg_) and the mean of the global efficiency of three nodes (T_eg_). To examine whether these two variables could be used to classify Westerners or Chinese participants, we conducted a prediction analysis based on logistic regression using the two combined variables as predictors and the cultural background (0 for Westerners and 1 for the Chinese individuals) as the dependent variable. Similarly, 10-fold cross-validation were conducted. The training set was used to generate a logistic regression model, which was further applied to the test set to generate predicted logits. With the cutoff set at 0.5, predicted logits higher than 0.5 were categorized as Chinese and those less than 0.5 were linked to Westerners. The predictive power was assessed by averaging the classification accuracies. Similarly, the statistical significance of the predictive power was determined through a permutation test. In this process, we randomly assigned Westerner and Chinese labels to each participant and repeated the 10-fold cross-validation. A null distribution of accuracy was generated through a permutation conducted 1000 times, and we evaluated whether the probability of obtaining the resulting accuracy was higher than the 95% CI of the null distribution.

##### Connectome-based predictive modeling.

Next, we adopted a method in functional brain imaging analysis—connectome-based predictive modeling (Finn *et al.*, 2015; Rosenberg *et al.*, 2016)—to test whether using the degree and global efficiency of all nodes to generate predictors would achieve a higher predictive power. We first conducted intrasample 10-fold cross-validation based on the degree and global efficiency of all nodes. In each iteration, the correlations between the nodal metrics (i.e. each node’s degree and global efficiency) and the AHS score were calculated based on the data from the training set. Nodal metrics significantly correlated with the AHS score (*P* < 0.05) were selected and further combined into two variables (T_deg_ and T_eg_) representing the means of degree and global efficiency, respectively. The two representative variables were then used to build a linear regression model for predicting the AHS score. The two combined variables from the test set were then extracted and entered into the model to generate the predicted AHS scores. The predictive power was also assessed by calculating the correlation between the predicted and observed AHS scores, and its significance level was also estimated by comparison to the null distribution generated by the permutation test.

Finally, we summarized the significant metrics in each iteration (in the previous step). Metrics that correlated with the AHS score 10 times were selected and combined into two variables (T_deg_ and T_eg_) using the data from Study 1 for each participant. Consistent with the previous cross-sample analysis, we conducted 10-fold cross-validation analysis using logistic regression, with the two merged variables as the predictors and the cultural background as the dependent variable. The procedures and the assessment of classification were the same as before.

##### Meta-analytic decoding.

To identify the psychological functions related to the important nodes in the prediction model of the holistic–analytic cultural style, we decoded the images of the important nodes and the remaining nodes using the Neurosynth Image Decoder (Yarkoni *et al.*, 2011). The Neurosynth Image Decoder enabled us to compare specific images to images associated with various psychological constructs in the Neurosynth database (version 0.7). The Neurosynth database contains the activation data for 14 371 studies and the feature information for over 3200 term-based features. Nodes were decoded by calculating the voxelwise Pearson correlation between the nodes’ image ﬁles and the meta-analytical image ﬁle belonging to each feature term.

## Results

**Fig. 3.**
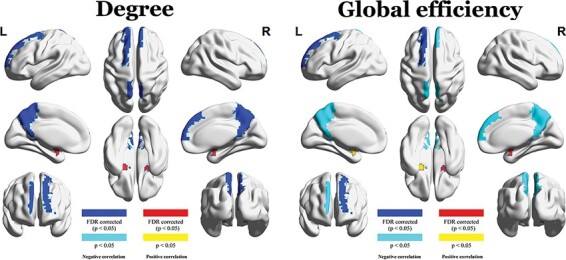
Partial correlations between the AUC of degree and global efficiency and the AHS.

Because Study 1 revealed consistent cultural differences in the metrics of 19 nodes, we further investigated whether the metrics were associated with holistic–analytic cognitive style across individuals in the Chinese sample. The partial correlation analyses revealed significant associations between the AHS score and 10 metrics (FDR corrected, *P* < 0.05). The degrees of the left SFGdor, right mPFC and bilateral PCUN were negatively correlated with the AHS score (*rs* = −0.20–(−0.15), *ps* = 1.27 × 10^−3^–0.02). The degrees of the bilateral AMYG and the left PUT were positively correlated with the AHS score (*rs* = 0.17–0.20, *ps* = 1.27 × 10^−3^–7.94 × 10^−3^; Figure 3, Table 3). Likewise, the global efficiency of the left SFGdor was negatively correlated with the AHS score (*r* = −0.20, *p* = 1.05 × 10^−3^). The global efficiency of the right AMYG and the left PUT were positively correlated with the AHS score (*rs* = 0.17, 0.17, *ps* = 5.19 × 10^−3^, 7.20 × 10^−3^; Figure 3, Table 3). We also conducted the partial correlation analyses following the supplementary analyses in Study 1 and found similar patterns (see Supplementary Tables S4–S6).

**Table 3.** Partial correlation between network metrics and the AHS

Nodes	Degree		Global efficiency	
	*r*	*P*	*R*	*P*
L.PreCG	−0.03	0.61	−0.03	0.66
R.PreCG	0.07	0.27	0.04	0.50
L.SFGdor	−0.20*	1.27 × 10^−3^	−0.20*	1.05 × 10^−3^
L.OLF	0.01	0.90	0.02	0.75
L.mPFC	−0.08	0.17	−0.10	0.12
R.mPFC	−0.16*	0.01	−0.15	0.02
R.HIP	0.11	0.08	0.06	0.30
R.PHG	−0.04	0.54	−0.04	0.47
L.AMYG	0.17*	7.94 × 10^−3^	0.16	0.01
R.AMYG	0.17*	5.40 × 10^−3^	0.17*	5.19 × 10^−3^
R.IOG	0.00	0.94	−0.03	0.63
L.PCUN	−0.15*	0.02	−0.15	0.02
R.PCUN	−0.15*	0.01	−0.12	0.05
L.PUT	0.20*	1.27 × 10^−3^	0.17*	7.20 × 10^−3^
R.PUT	0.13	0.04	0.10	0.10
L.PAL	0.13	0.04	0.11	0.07
R.PAL	0.00	0.96	0.03	0.61
R.TPOsup	0.12	0.07	0.11	0.08
L.TPOmid	0.05	0.40	0.10	0.10

Abbreviations: L. PreCG, left precental gyrus; R. PreCG, right precental gyrus; L. SFGdor, left dorsal superior frontal gyrus; L. OLF, left olfactory cortex; L. mPFC, left medial prefrontal cortex; R. mPFC, right medial prefrontal cortex; R. HIP, right hippocampus; R. PHG, right parahippocampal gyrus; L. AMYG, left amygdala; R. AMYG, right amygdala; R. IOG, right inferior occipital gyrus; L. PCUN, left precuneus; R. PCUN, right precuneus; L. PUT, left putamen; R. PUT, right putamen; L. PAL, left pallidum; R. PAL, right pallidum; R. TPOsup, right superior temporal pole; L. TPOmid, left middle temporal pole.

* *P* < 0.05, FDR corrected.

**Fig. 4.**
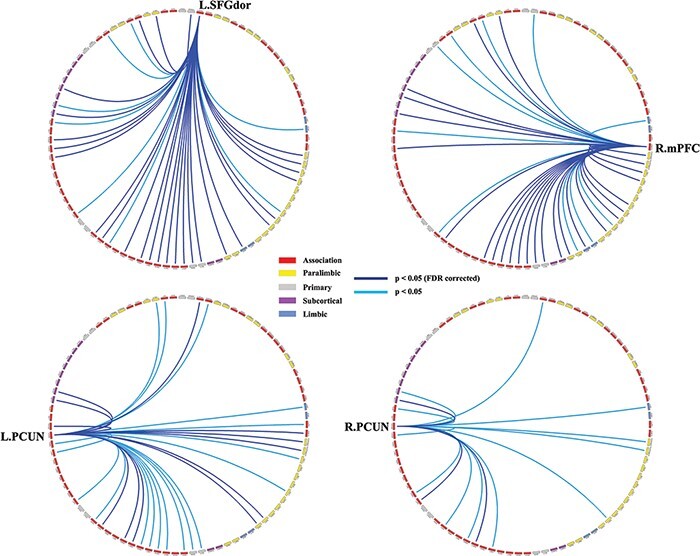
Partial correlations between functional connectivity and the AHS. Abbreviations: L. SFGdor, left dorsal superior frontal gyrus; R. AMYG, right amygdala; L. PCUN, left precuneus; R. PCUN, right precuneus. Please see Supplementary Table S11 for the abbreviations and the functional classification of all nodes.

We further tested whether these nodes’ functional connectivity with the other 89 nodes was associated with the holistic thinking style. The results (FDR corrected, *P* < 0.05) showed that the functional connectivity strength from the left SFGdor to 27 nodes showed significant negative correlations with the AHS score (*rs* = −0.24–(−0.15), *ps* = 3.90 × 10^−5^–0.01; Figure 4, Supplementary Table S7). Similarly, functional connectivity between the right mPFC and 26 nodes (*rs*= −0.24–(−0.15), *ps *= 6.00 × 10^−5^< *ps* < 0.01; Figure 4, supplementary results Table S8), functional connectivity between the left PCUN and 12 nodes (*rs*= −0.21–(−0.16), *ps *= 2.35 × 10^−4^ < *ps* < 6.64 × 10^−3^; Figure 4, Supplementary Table S9) and functional connectivity between the right PCUN to 3 nodes (*rs* = −0.21–(−0.19), *ps* = 5.27 × 10^−4^–1.53 × 10^−3^; Figure 4, Supplementary Table S10) was also negatively correlated with the AHS score. There was no significant correlation between the AHS score and the functional connectivity of the bilateral AMYG or left PUT.

**Fig. 5.**
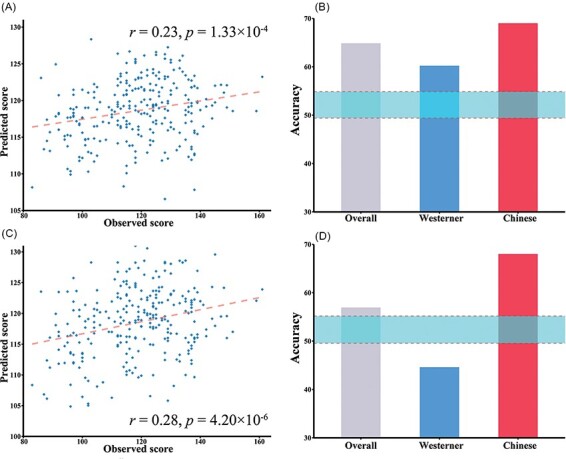
Results of the intrasample and cross-sample K-fold cross-validation analyses. A) The correlation between the observed and predicted scores in intrasample cross-validation based on 10 metrics; B) the classification accuracies in cross-sample cross-validation based on 10 metrics; C) the correlation between the observed and predicted scores in intrasample cross-validation involving all nodal metrics; D) the classification accuracies in cross-sample cross-validation involving all nodal metrics. Blue ribbons in b and d represent the 95% CI generated by permutation tests.

We found that seven nodes’ AUCs for degree and three nodes’ AUCs for global efficiency were significantly correlated with the AHS score. We then examined whether these metrics could be used as effective predictors for holistic thinking style or cultural background. We merged the AUCs for the degree of the left SFGdor, right mPFC, bilateral PCUN, bilateral AMYG and left PUT and the AUCs for the global efficiency of the left SFGdor, right AMYG and left PUT into two combined variables in the datasets of Study 1 and Study 2. In the intrasample 10-fold cross-validation analysis, the correlation between the predicted and observed scores was significant (*r* = 0.23, *P* = 1.33 × 10^−4^; Figure 5A). The permutation test revealed a null distribution of the correlation coefficients with a 95% CI [−0.22, 0.12], indicating that the predictive power was significantly higher than the chance level. In the cross-sample validation analysis, we used 10-fold cross-validation based on logistic regression analysis, and the overall accuracy was 64.9% (60.22% of the Westerners and 69.00% of the Chinese participants were classified correctly). The permutation test revealed a null distribution of the overall accuracy, with a 95% CI [49.4%, 54.8%], which indicated that the obtained overall accuracy of the 10-fold cross-validation was significant (Figure 5B).

In the validation analyses that adopted the method from connectome-based predictive modeling (Finn *et al.*, 2015; Rosenberg *et al.*, 2016) and involved all node metrics (without prior knowledge), intrasample 10-fold cross-validation showed that the correlation between the observed and predicted scores was significant (*r* = 0.28, *P* = 4.20 × 10^−6^; Figure 5C). The 95% CI of the null distribution of the predictive power was [−0.31, 0.15], indicating that the obtained predictive power was significantly higher than the chance level. Using Fisher r-to-z transformation, we found no significant difference between the current predictive power and the former predictive power based on 10 metrics (*P* = 0.29). In the cross-sample cross-validation, the degree of 17 nodes and the global efficiency of 15 nodes, which were correlated with the AHS score 10 times in the previous step, were used to generate the predictors for the logistic regression model using the data from Study 1. The total accuracy was 56.93% (44.60% of the Westerners and 68.00% of the Chinese participants were categorized correctly). The permutation test revealed a null distribution of the overall accuracy, with a 95% CI [49.5%, 55.1%], indicating that the obtained overall accuracy was significantly higher than the chance level (Figure 5D).

**Fig. 6.**
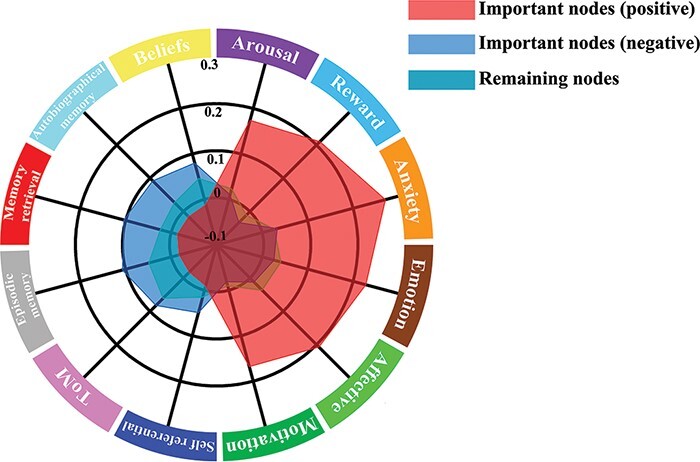
Meta-decoding results of important nodes in the prediction model of holistic–analytic cultural style.

Meta-analytic decoding found that the important nodes positively predicting holistic–analytic cultural style were more related to psychological constructs associated with reward and affective function, including the categories ‘anxiety’, ‘reward’, ‘emotion’, ‘affective’, ‘motivation’ and ‘arousal’, and the important nodes negatively predicting holistic–analytic cultural style were more related to psychological constructs associated with self and mind, including the categories ‘autobiographical memory’, ‘episodic memory’, ‘beliefs’, ‘ToM (Theory of Mind)’, ‘memory retrieval’ and ‘self-referential’ than the other nodes were (Figure 6).

## Discussion

In Study 1, by measuring the degree and global efficiency, we discovered that culture affects the cerebral information integration function of the nodes in the DMN, AMYG and basal ganglia. Specifically, nodes in the DMN are more crucial in the brain networks of Westerners, while nodes in the subcortical area (i.e. basal ganglia, AMYG) are more important for Chinese individuals. In Study 2, we further established the connection between culturally different nodal graph metrics and holistic thinking style. Metrics of nodes in the DMN (i.e. mPFC, PCUN) were negatively correlated with holistic thinking style, whereas metrics of the nodes in subcortical regions (i.e. AMYG, PUT) were positively correlated with holistic thinking style. Moreover, we discovered that the functional connectivity between the nodes in the DMN and other nodes in the brain network were negatively correlated with holistic thinking style. Importantly, the metrics that were correlated with holistic thinking style can be viewed as neural representations of culture and can be used as predictors for holistic thinking style and cultural background. Based on these two studies, we can summarize that people from the East and West exhibit diverse organizational characteristics in their functional brain networks. The topological features of the brain network are associated with specific cultural values and can be viewed as neural representations as well as effective predictors of culture.

Consistent with our hypotheses, we discovered that the nodes in the DMN are crucial for Westerner’s functional brain network. This result is in line with previous research that has posited a relationship between interdependence/independence and the gray-matter volume of DMN regions. For example, there is a negative correlation between interdependence and the gray-matter volume of the orbitofrontal cortex (Kitayama *et al.*, 2017) and a positive correlation between independence and the gray-matter volume of the vmPFC, right DLPFC and right RLPFC (Wang *et al.*, 2017). As noted by Wang *et al.* (2017), these brain regions are related to the function of self-agency. Using R-fMRI and graph theoretical analysis, our findings further support this notion by showing that the functional properties of the DMN in the brain network are more important for Westerners. This result could be explained by the connection between the DMN and self-consciousness, which has been shown in previous research. It was found that the DMN is strongly connected to an individual’s personality (Lei *et al.*, 2013) and overlaps with the self-reference network (Legrand and Ruby, 2009; Qin and Northoff, 2011; Whitfield-Gabrieli *et al.*, 2011). Furthermore, important regions in the DMN, such as the posterior cingulate cortex and PCUN, have been considered key components of the neural network correlates of consciousness (Vogt and Laureys, 2005) and impact self-related and episodic memory tasks (Cavanna and Trimble, 2006; Raichle, 2015). The higher priority of DMN nodes in the brain network may help Westerners maintain their independent self-construal.

In contrast, we discovered different functional features among Chinese individuals. As hypothesized, we discovered that AMYG plays a crucial role in the brain networks of Chinese individuals. One potential reason is that interdependent East Asians are more willing to maintain harmonious relationships with others (Markus and Kitayama, 1991), and the processing of others’ emotions may promote the achievement of this goal (Tsai *et al.*, 2007). Research has found that, compared to people from Western cultural backgrounds, East Asians are more accurate when inferring the emotions of close others (Ma-Kellams and Blascovich, 2012) and more sensitive to the disappearance of positive emotions rather than negative emotions (Ishii *et al.*, 2011). Their AMYG also shows greater activation when participating in emotional tasks (Derntl *et al.*, 2012). Thus, the critical role of the AMYG in the resting-state functional network may represent the characteristics or strategies of socioemotional processing for East Asians. Another explanation is that East Asians are more likely to regulate their emotions by suppressing their emotions (Matsumoto *et al.*, 2008), and the AMYG contributes to emotion regulation (Hare *et al.*, 2005; Banks *et al.*, 2007; Frank *et al.*, 2014). In addition to the hypothesized results concerning the AMYG, we found that nodes in the basal ganglia (i.e. PUT and PAL) are also important in the brain networks of Chinese individuals. Inside the basal ganglia, the striatum has been argued to make critical contributions to emotion regulation (Hare *et al.*, 2005), and meta-analysis research revealed the involvement of the PUT and PAL in this process (Kohn *et al.*, 2014). Thus, the characteristics of emotion processing and emotion regulation may explain the importance of the AMYG and basal ganglia in the functional brain networks of Chinese individuals. Notably, we also found that Chinese individuals exhibited a significantly higher degree and global efficiency in the temporal pole (i.e. TPOsup and TPOmid). Although the temporal pole belongs to the DMN (Andrews-Hanna *et al.*, 2010) and participates in self-reference tasks (Longe *et al.*, 2010; Pauly *et al.*, 2014), evidence also shows that the temporal pole is crucial for representing and retrieving social knowledge (Olson *et al.*, 2013), integrating social information (Pehrs *et al.*, 2017), making attributions based on contexts (Kestemont *et al.*, 2015), theory of mind (Ross and Olson, 2010) and emotion regulation (Taylor *et al.*, 2018). It is possible that the temporal pole cooperates with the AMYG and basal ganglia in processing socioemotional information, which increases its importance in the brain networks of Chinese individuals.

Study 1 revealed the general cultural differences in the organizational characteristics of the functional brain network, and in Study 2, we further illustrated that the cultural differences can be partly explained by thinking style. The nodal metrics in the DMN (i.e. mPFC and PCUN), basal ganglia (i.e. left PUT) and AMYG were correlated with a holistic–analytic thinking style. As discussed, DMN regions are responsible for self-agency. Therefore, the association between DMN nodal metrics and analytic thinking may suggest that independent self-construal is strongly associated with analytic thinking style, which has been found in experimental research (Krishna *et al.*, 2008; Kitayama *et al.*, 2009; Lin and Han, 2009; Lalwani and Shavitt, 2013). For instance, priming independent or interdependent self-knowledge induces the corresponding thinking style (analytic or holistic thinking; Kühnen *et al.*, 2001; Monga and John, 2010). The positive correlations between holistic thinking style and the nodal metrics of the AMYG and left PUT may imply that the processing of socioemotional information or the regulation of self-moods may require abilities that rely on holistic thinking. Integrating social information and switching attention to different social targets may be related to the function of changing the ‘locus of attention’, which is a main construct in holistic–analytic thinking.

We also found that the functional connectivity between the nodes in the DMN (mPFC and PCUN) to other nodes was negatively correlated with the holistic thinking style, that is, functional connectivity was positively correlated with the analytic thinking style. We suggest that the functional connectivity of the mPFC and PCUN may fall into two categories and can be interpreted in two different ways. First, the negative correlations between intranetwork connectivity (functional connectivity between the mPFC and PCUN and other nodes in the DMN) and holistic thinking style properly represent the relationship between the intranetwork connectivity of the DMN and self-concept. This view is in line with the findings from psychiatry studies. In psychological diseases such as depression, posttraumatic stress disorder and schizophrenia, which are characterized by rumination and increased self-focused thoughts (Ingram, 1990; Watkins and Teasdale, 2001; Speckens *et al.*, 2007), increased intranetwork connectivity of the DMN was observed (Greicius *et al.*, 2007; Whitfield-Gabrieli *et al.*, 2009; Dunkley *et al.*, 2015). Second, the negative correlations between the internetwork connectivity (functional connectivity between the mPFC and PCUN and other nodes outside the DMN) and holistic thinking style can be viewed as the redistribution of resources in the brain networks of East Asians, who generally do not have the mindset of independence or analytic thinking as the ‘default mode’. East Asians may need to inhibit the internetwork connectivity of the DMN and allocate more resources to intranetwork connectivity, through which intranetwork connectivity can be enhanced and facilitate the processing of information in a way analogous to Western brain networks.

Taken together, the different organizational features of the brain network across cultural groups and the association between thinking style and the metrics of the DMN and subcortical areas demonstrate that culture and brain network properties are closely linked (Kitayama and Uskul, 2011; Han and Ma, 2015). Specifically, our findings are aligned with the perspectives from the culture–behavior–brain (CBB) loop model (Han and Ma, 2015) and the neuro-culture interaction model (Kitayama and Uskul, 2011). Both models suggest that due to the plasticity of the brain, repeatedly engaging in tasks or conducting behaviors related to a specific cultural context may promote the formation of culturally patterned neural activities, which may further facilitate the generation of culturally related behaviors or adaptive actions (Kitayama and Uskul, 2011; Han and Ma, 2015). Additionally, through intrasample and cross-sample validation analyses, we discovered that functional metrics are efficient predictors of an individual’s cultural background and the tendency toward holistic–analytic thinking. Thus, functional graph metrics are not only strongly associated with culture but can also be viewed as ‘neuromarkers’ or functional connectome fingerprints of culture and a holistic–analytic thinking style.

This study has some limitations that could be addressed in future research. First, the public datasets do not provide detailed demographic information about the sample and do not provide measurements related to cultural values, which restricts the ecological validity of our findings. Further research involving detailed measurements will be helpful in verifying our findings. In addition, data for Study 1 were collected from multiple centers, which raises the possibility that differences in scanning (e.g. scan parameters, environments) may confound the findings. Likewise, as age is an essential factor that affects the segregation of brain networks (Chan *et al.*, 2014), differences in age across Western and Chinese samples may also compromise ecological validity. More rigorous cross-cultural and multiple-center R-fMRI studies will be helpful in assessing the robustness of the findings of Study 1. Second, we discovered significant correlations only between holistic–analytic thinking style and the metrics of seven nodes. As culture is a complex system (Jahoda, 2012), whether those metrics that were not significantly correlated with holistic thinking style are correlated with other cultural subdimensions remains unknown. In addition, we also found higher accuracies for Chinese participants compared to Westerners in cross-validation analyses for predicting cultural groups. One possible reason is that the predictors that we used are generated from our Chinese sample. Additional research is necessary to explore whether culture moderates the associations between brain topological properties and holistic–analytic thinking style. Moreover, as the CBB loop model (Han and Ma, 2015) proposed, culture and the brain may be connected through culturally related behaviors. More research is required to disentangle the relationships between functional network properties and culture. Similarly, the associations between the graph metrics and holistic–analytic thinking do not determine the individuals’ behaviors in specific tasks that involve holistic–analytic thinking. Thus, a possible research direction is to examine whether R-fMRI network properties are efficient predictors participant behaviors or performance on culturally related tasks.

In conclusion, by building up R-fMRI networks and using graph theoretical analysis, our research explored the cultural effect on the topological properties of the brain and discovered the culturally patterned characteristics of brain networks. We also provided a possible explanation for the link between brain networks and culture by revealing the correlations between global properties and the holistic–analytic thinking style. More importantly, functional graph metrics can be regarded as fingerprints of our cultural background and cultural tendencies, which enriches the current understanding of the relationship between culture and the brain.

## Supplementary Material

nsab080_Supp
