# Supplementary material for: Functional connectome fingerprint of holistic–analytic cultural style
Source: Soc Cogn Affect Neurosci. 2021 Jun 23;17(2):172–86. doi: 10.1093/scan/nsab080 (PMC8847908; doi:10.1093/scan/nsab080)
Supplement: nsab080_Supp [file nsab080_supp.zip › Supplementary materials.docx]

**Supplementary materials**

**Functional** **Connectome Fingerprint of** **Holistic-Analytic Cultural Style**

**Siyang Luo^1^**^*^**, Yiyi Zhu^1,3^**^*^**, Shihui Han^2^**

**^1^Department of Psychology,**

**Guangdong Key Laboratory of Social Cognitive Neuroscience and Mental Health**

**Guangdong Provincial Key Laboratory of Brain Function and Disease**

**Sun Yat-sen University, Guangzhou 510006, China**

**^2^School of Psychological and Cognitive Sciences**

**PKU-IDG/McGovern Institute for Brain Research**

**Beijing Key Laboratory of Behavior and Mental Health**

**Peking University, Beijing 100080, China**

**^3^School of Behavioral and Brain Sciences,**

**The University of Texas at Dallas, Richardson, TX 75080, United States**

^*^ **The first two authors contributed equally to this work.**

**Running title: Connectome and culture**

Correspondence should be addressed to:

Siyang Luo, Ph.D.

Department of Psychology

Sun Yat-Sen University, Guangzhou 510006, China

Email: ljc520ida@163.com

**Table S1.** *Nodes Identified Using MANCOVA and ANCOVA Analyses of the AUC for the Degree and Global Efficiency Based on Data That Regressed out the Global Signals*

| Node | | MANCOVA | | | ANCOVA of Degree | | | | ANCOVA of Global efficiency | | | |
| --- | --- | --- | --- | --- | --- | --- | --- | --- | --- | --- | --- | --- |
|  |  | *F* | *p* | *η_p_^2^* | *F* | *p* | *η_p_^2^* | West/East | *F* | *p* | *η_p_^2^* | West/East |
| Right dorsal superior frontal gyrus | R.SFGdor | 9.02 | 1.39×10^-4^ | .031 | 17.81 | 2.84×10^-5^ | .031 | W | 11.19 | 8.79×10^-4^ | .020 | W |
| Left middle frontal gyrus | L.MFG | 8.92 | 1.54×10^-4^ | .031 | 17.8 | 2.86×10^-5^ | .031 | W | 12.41 | 4.61×10^-4^ | .022 | W |
| Right middle frontal gyrus | R.MFG | 7.90 | 4.13×10^-4^ | .028 | 13.29 | 2.92×10^-4^ | .023 | W | 15.69 | 8.43×10^-5^ | .027 | W |
| Right inferior frontal gyrus (triangular part) | R.IFGtriang | 13.13 | 2.69×10^-6^ | .045 | 14.33 | 1.70×10^-4^ | .025 | W | 24.21 | 1.14×10^-6^ | .042 | W |
| Left rolandic operculum | L.ROL | 7.62 | 5.43×10^-4^ | .027 | 15.12 | 1.13×10^-4^ | .026 | E | 9.10 | .003 | .016 | E |
| Right rolandic operculum | R.ROL | 13.34 | 2.19×10^-6^ | .046 | 26.14 | 4.36×10^-7^ | .045 | E | 13.70 | 2.36×10^-4^ | .024 | E |
| Right supplementary motor area | R.SMA | 8.65 | 1.99×10^-4^ | .030 | 17.34 | 3.63×10^-5^ | .030 | E | 13.68 | 2.39×10^-4^ | .024 | E |
| Left olfactory cortex | L.OLF | 5.98 | .003 | .021 | 11.98 | 5.80×10^-4^ | .021 | E | 10.44 | .001 | .018 | E |
| Right olfactory cortex | R.OLF | 5.15 | .006 | .018 | 9.97 | .002 | .018 | E | 7.22 | .007 | .013 | E |
| Left superior frontal gyrus, medial orbital | L.ORBsupmed | 3.73 | .025 | .013 | 7.44 | .007 | .013 | E | 6.09 | .014 | .011 | E |
| Right superior frontal gyrus, medial orbital | R.ORBsupmed | 3.69 | .026 | .013 | 6.46 | .011 | .011 | E | 7.19 | .008 | .013 | E |
| Right gyrus rectus | R.REC | 6.49 | .002 | .023 | 12.52 | 4.36×10^-4^ | .022 | E | 11.91 | 6.01×10^-4^ | .021 | E |
| Left hippocampus | L.HIP | 7.30 | 7.44×10^-4^ | .026 | 13.73 | 2.32×10^-4^ | .024 | W | 14.21 | 1.81×10^-4^ | .025 | W |
| Left amygdala | L.AMYG | 9.18 | 1.19×10^-4^ | .032 | 18.02 | 2.56×10^-5^ | .031 | E | 16.94 | 4.45×10^-5^ | .029 | E |
| Right amygdala | R.AMYG | 12.48 | 4.99×10^-6^ | .043 | 24.99 | 7.72×10^-7^ | .043 | E | 19.97 | 9.54×10^-6^ | .035 | E |
| Left cuneus | L.CUN | 7.00 | 9.97×10^-4^ | .025 | 6.94 | .009 | .012 | W | 13.83 | 2.20×10^-4^ | .024 | W |
| Right cuneus | R.CUN | 10.30 | 4.04×10^-5^ | .036 | 16.94 | 4.45×10^-5^ | .029 | W | 18.88 | 1.66×10^-5^ | .033 | W |
| Left superior occipital gyrus | L.SOG | 9.52 | 8.60×10^-5^ | .033 | 14.74 | 1.38×10^-4^ | .026 | W | 18.07 | 2.50×10^-5^ | .031 | W |
| Right superior occipital gyrus | R.SOG | 11.87 | 8.91×10^-6^ | .041 | 21.35 | 4.75×10^-6^ | .037 | W | 20.09 | 8.95×10^-6^ | .035 | W |
| Left middle occipital gyrus | L.MOG | 5.66 | .004 | .020 | 5.35 | .021 | .009 | W | 11.14 | 8.99×10^-4^ | .02 | W |
| Right middle occipital gyrus | R.MOG | 10.22 | 4.38×10^-5^ | .035 | 17.86 | 2.77×10^-5^ | .031 | W | 18.25 | 2.28×10^-5^ | .032 | W |
| Left superior parietal gyrus | L.SPG | 6.26 | .002 | .022 | 9.70 | .002 | .017 | W | 12.52 | 4.36×10^-4^ | .022 | W |
| Right angular gyrus | R.ANG | 11.38 | 1.43×10^-5^ | .039 | 14.03 | 1.99×10^-4^ | .025 | W | 22.60 | 2.54×10^-6^ | .039 | W |
| Left precuneus | L.PCUN | 11.59 | 1.17×10^-5^ | .040 | 21.12 | 5.35×10^-6^ | .036 | W | 22.20 | 3.11×10^-6^ | .038 | W |
| Right precuneus | R.PCUN | 8.14 | 3.28×10^-4^ | .028 | 14.80 | 1.34×10^-4^ | .026 | W | 15.56 | 9.02×10^-5^ | .027 | W |
| Left putamen | L.PUT | 10.68 | 2.80×10^-5^ | .037 | 19.92 | 9.75×10^-6^ | .034 | E | 12.43 | 4.56×10^-4^ | .022 | E |
| Right putamen | R.PUT | 14.56 | 6.84×10^-7^ | .050 | 26.93 | 2.96×10^-7^ | .046 | E | 15.70 | 8.38×10^-5^ | .027 | E |
| Left superior temporal gyrus | L.STG | 4.77 | .009 | .017 | 9.49 | .002 | .017 | E | 7.72 | .006 | .014 | E |
| Left superior temporal pole | L.TPOsup | 6.82 | .001 | .024 | 13.65 | 2.42×10^-4^ | .024 | E | 12.26 | 5.01×10^-4^ | .021 | E |
| Right superior temporal pole | R.TPOsup | 10.70 | 2.76×10^-5^ | .037 | 21.15 | 5.25×10^-6^ | .037 | E | 16.09 | 6.86×10^-5^ | .028 | E |
| Left middle temporal gyrus | L.MTG | 5.83 | .003 | .021 | 10.59 | .001 | .019 | W | 11.21 | 8.69×10^-4^ | .020 | W |
| Right middle temporal gyrus | R.MTG | 4.35 | .013 | .015 | 5.54 | .019 | .010 | W | 8.36 | .004 | .015 | W |

*Note*. W represents a significantly higher value among Westerners; E represents a significantly higher value among East Asians (i.e., Chinese individuals).

**Table S2.** *Nodes Identified Using MANCOVA and ANCOVA Analyses of the AUC for the Degree and Global Efficiency Calculated Based on an Absolute Network*

| Node | | MANCOVA | | | ANCOVA of Degree | | | | ANCOVA of Global efficiency | | | |
| --- | --- | --- | --- | --- | --- | --- | --- | --- | --- | --- | --- | --- |
|  |  | *F* | *p* | *η_p_^2^* | *F* | *p* | *η_p_^2^* | West/East | *F* | *p* | *η_p_^2^* | West/East |
| Left precental gyrus | L.PreCG | 6.92 | .001 | .024 | 13.65 | 2.41×10^-4^ | .024 | W | 10.97 | 9.87×10^-4^ | .019 | W |
| Right precental gyrus | R.PreCG | 6.00 | .003 | .021 | 11.93 | 5.94×10^-4^ | .021 | W | 9.05 | .003 | .016 | W |
| Left dorsal superior frontal gyrus | L.SFGdor | 8.04 | 3.62×10^-4^ | .028 | 14.14 | 1.87×10^-4^ | .025 | W | 8.47 | .004 | .015 | W |
| Left olfactory cortex | L.OLF | 6.95 | .001 | .024 | 7.83 | .005 | .014 | E | 13.69 | 2.37×10^-4^ | .024 | E |
| Left medial prefrontal cortex | L.mPFC | 7.17 | 8.46×10^-4^ | .025 | 13.44 | 2.70×10^-4^ | .024 | W | 9.32 | .002 | .016 | W |
| Right medial prefrontal cortex | R.mPFC | 6.22 | .002 | .022 | 11.98 | 5.78×10^-4^ | .021 | W | 8.66 | .003 | .015 | W |
| Right hippocampus | R.HIP | 5.70 | .004 | .020 | 11.17 | 8.89×10^-4^ | .020 | E | 6.82 | .009 | .012 | E |
| Right parahippocampal gyrus | R.PHG | 5.26 | .005 | .019 | 8.09 | .005 | .014 | E | 10.53 | .001 | .019 | E |
| Left amygdala | L.AMYG | 10.15 | 4.70×10^-5^ | .035 | 12.76 | 3.85×10^-4^ | .022 | E | 20.13 | 8.81×10^-6^ | .035 | E |
| Right amygdala | R.AMYG | 16.28 | 1.35×10^-7^ | .055 | 25.31 | 6.58×10^-7^ | .043 | E | 32.59 | 1.85×10^-8^ | .055 | E |
| Left precuneus | L.PCUN | 17.73 | 3.43×10^-8^ | .060 | 34.60 | 6.97×10^-9^ | .058 | W | 24.91 | 8.02×10^-7^ | .043 | W |
| Right precuneus | R.PCUN | 11.96 | 8.24×10^-6^ | .041 | 22.87 | 2.22×10^-6^ | .039 | W | 15.42 | 9.70×10^-5^ | .027 | W |
| Left putamen | L.PUT | 8.75 | 1.82×10^-4^ | .030 | 13.07 | 3.27×10^-4^ | .023 | E | 17.53 | 3.29×10^-5^ | .030 | E |
| Right putamen | R.PUT | 10.18 | 4.56×10^-5^ | .035 | 13.21 | 3.04×10^-4^ | .023 | E | 20.16 | 8.67×10^-6^ | .035 | E |
| Left pallidum | L.PAL | 19.93 | 4.37×10^-9^ | .067 | 38.16 | 1.26×10^-9^ | .064 | E | 35.98 | 3.59×10^-9^ | .061 | E |
| Right pallidum | R.PAL | 21.80 | 7.65×10^-10^ | .073 | 41.53 | 2.51×10^-10^ | .069 | E | 38.80 | 9.26×10^-10^ | .065 | E |
| Right superior temporal pole | R.TPOsup | 7.05 | 9.46×10^-4^ | .025 | 9.31 | .002 | .016 | E | 13.52 | 2.58×10^-4^ | .024 | E |
| Left middle temporal pole | L.TPOmid | 14.34 | 8.50×10^-7^ | .049 | 23.06 | 2.02×10^-6^ | .040 | E | 28.35 | 1.47×10^-7^ | .048 | E |

*Note*. W represents a significantly higher value among Westerners; E represents a significantly higher value among East Asians (i.e., Chinese individuals).

**Table S3.** *Nodes Identified Using MANCOVA and ANCOVA Analyses of the AUC for the Degree and Global Efficiency Based on Data Using the Power 264 Template*

|  |  |  | MANCOVA | | | ANCOVA of Degree | | | | ANCOVA of Global efficiency | | | |
| --- | --- | --- | --- | --- | --- | --- | --- | --- | --- | --- | --- | --- | --- |
| Node | Suggested network | *MNI* | *F* | *p* | *η_p_^2^* | *F* | *p* | *η_p_^2^* | West/East | *F* | *p* | *η_p_^2^* | West/East |
| 10 | Uncertain | [52, 34, 27] | 11.76 | 9.98×10^-6^ | .041 | 22.04 | 3.36×10^-6^ | .038 | E | 19.99 | 9.45×10^-6^ | .035 | E |
| 11 | Uncertain | [55, 31, 17] | 11.70 | 1.05×10^-5^ | .04 | 23.24 | 1.84×10^-6^ | .040 | E | 19.15 | 1.44×10^-5^ | .033 | E |
| 24 | Sensory/somatomotor Hand | [-40, 19,54] | 6.88 | .001 | .024 | 12.38 | 4.69×10^-4^ | .022 | W | 13.79 | 2.25×10^-4^ | .024 | W |
| 27 | Sensory/somatomotor Hand | [-38, 27,69] | 12.48 | 5.01×10^-6^ | .043 | 24.07 | 1.22×10^-6^ | .041 | W | 23.60 | 1.55×10^-6^ | .041 | W |
| 30 | Sensory/somatomotor Hand | [-29, 43,61] | 5.13 | .006 | .018 | 10.27 | .001 | .018 | E | 9.25 | .002 | .016 | E |
| 31 | Sensory/somatomotor Hand | [10, 17,74] | 4.62 | .010 | .016 | 6.86 | .009 | .012 | E | 9.14 | .003 | .016 | E |
| 39 | Sensory/somatomotor Hand | [2, 28,60] | 10.93 | 2.22×10^-5^ | .038 | 19.57 | 1.17×10^-5^ | .034 | W | 21.89 | 3.62×10^-6^ | .038 | W |
| 40 | Sensory/somatomotor Hand | [3, 17,58] | 4.75 | .009 | .017 | 7.86 | .005 | .014 | W | 9.32 | .002 | .016 | W |
| 46 | Sensory/somatomotor Mouth | [66, 8,25] | 4.70 | .009 | .017 | 8.29 | .004 | .015 | W | 9.21 | .003 | .016 | W |
| 49 | Cingulo-opercular Task Control | [19, 8,64] | 7.14 | 8.68×10^-4^ | .025 | 14.00 | 2.01×10^-4^ | .024 | E | 9.09 | .003 | .016 | E |
| 61 | Auditory | [32, 26,13] | 8.55 | 2.20×10^-4^ | .030 | 14.07 | 1.95×10^-4^ | .025 | W | 17.12 | 4.06×10^-5^ | .03 | W |
| 63 | Auditory | [58, 16,7] | 6.47 | .002 | .023 | 12.95 | 3.49×10^-4^ | .023 | W | 11.58 | 7.14×10^-4^ | .02 | W |
| 64 | Auditory | [-38, 33,17] | 22.42 | 4.30×10^-10^ | .075 | 42.80 | 1.37×10^-10^ | .071 | W | 42.02 | 1.99×10^-10^ | .07 | W |
| 66 | Auditory | [-49, 26,5] | 5.30 | .005 | .019 | 10.13 | .002 | .018 | W | 10.45 | .001 | .018 | W |
| 67 | Auditory | [43, 23,20] | 9.54 | 8.46×10^-5^ | .033 | 16.50 | 5.58×10^-5^ | .029 | W | 18.57 | 1.94×10^-5^ | .032 | W |
| 73 | Auditory | [-30, 27,12] | 5.78 | .003 | .020 | 7.76 | .006 | .014 | W | 11.29 | 8.31×10^-4^ | .02 | W |
| 77 | Default mode | [-13, 40,1] | 12.28 | 6.07×10^-6^ | .042 | 24.50 | 9.87×10^-7^ | .042 | W | 19.35 | 1.30×10^-5^ | .034 | W |
| 82 | Default mode | [46,16, 30] | 7.26 | 7.72×10^-4^ | .025 | 13.00 | 3.39×10^-4^ | .023 | E | 13.88 | 2.14×10^-4^ | .024 | E |
| 88 | Default mode | [-7, 55,27] | 7.50 | 6.11×10^-4^ | .026 | 6.34 | .012 | .011 | W | 11.36 | 8.03×10^-4^ | .02 | W |
| 89 | Default mode | [6, 59,35] | 4.87 | .008 | .017 | 7.23 | .007 | .013 | W | 9.38 | .002 | .017 | W |
| 90 | Default mode | [-11, 56,16] | 5.59 | .004 | .020 | 9.70 | .002 | .017 | W | 11.20 | 8.74×10^-4^ | .02 | W |
| 91 | Default mode | [-3, 49,13] | 6.05 | .003 | .021 | 11.72 | 6.64×10^-4^ | .021 | W | 8.66 | .003 | .015 | W |
| 93 | Default mode | [15, 63,26] | 13.41 | 2.06×10^-6^ | .046 | 25.27 | 6.74×10^-7^ | .043 | W | 26.59 | 3.50×10^-7^ | .045 | W |
| 95 | Default mode | [11, 54,17] | 9.96 | 5.61×10^-5^ | .035 | 13.90 | 2.12×10^-4^ | .024 | W | 18.95 | 1.60×10^-5^ | .033 | W |
| 103 | Default mode | [-10,55,39] | 7.75 | 4.79×10^-4^ | .027 | 11.23 | 8.58×10^-4^ | .020 | W | 15.41 | 9.73×10^-5^ | .027 | W |
| 106 | Default mode | [6,64,22] | 7.93 | 4.00×10^-4^ | .028 | 14.76 | 1.36×10^-4^ | .026 | W | 15.53 | 9.16×10^-5^ | .027 | W |
| 107 | Default mode | [-7,51, 1] | 7.46 | 6.37×10^-4^ | .026 | 14.71 | 1.40×10^-4^ | .026 | W | 14.11 | 1.91×10^-4^ | .025 | W |
| 109 | Default mode | [-3,44, 9] | 13.02 | 2.97×10^-6^ | .045 | 20.58 | 7.02×10^-6^ | .036 | W | 25.62 | 5.66×10^-7^ | .044 | W |
| 123 | Default mode | [52, 2, 16] | 4.60 | .010 | .016 | 8.24 | .004 | .015 | E | 8.96 | .003 | .016 | E |
| 124 | Default mode | [-26, 40, 8] | 10.25 | 4.26×10^-5^ | .035 | 17.21 | 3.86×10^-5^ | .030 | W | 20.11 | 8.88×10^-6^ | .035 | W |
| 126 | Default mode | [-34, 38, 16] | 5.02 | .007 | .018 | 9.11 | .003 | .016 | W | 9.33 | .002 | .016 | W |
| 144 | Visual | [40, 72,14] | 4.57 | .011 | .016 | 6.87 | .009 | .012 | E | 8.93 | .003 | .016 | E |
| 147 | Visual | [-28, 79,19] | 6.61 | .001 | .023 | 9.83 | .002 | .017 | E | 13.08 | 3.25×10^-4^ | .023 | E |
| 160 | Visual | [-16, 52, 1] | 12.31 | 5.86×10^-6^ | .042 | 23.61 | 1.54×10^-6^ | .041 | W | 23.38 | 1.72×10^-6^ | .04 | W |
| 163 | Visual | [6, 72,24] | 6.10 | .002 | .021 | 10.44 | .001 | .018 | W | 12.13 | 5.35×10^-4^ | .021 | W |
| 184 | Uncertain | [17, 80, 34] | 5.22 | .006 | .018 | 8.89 | .003 | .016 | E | 10.22 | .001 | .018 | E |
| 185 | Uncertain | [35, 67, 34] | 7.17 | 8.40×10^-4^ | .025 | 13.28 | 2.94×10^-4^ | .023 | E | 6.34 | .012 | .011 | E |
| 196 | Fronto-parietal Task Control | [40,18,40] | 7.56 | 5.77×10^-4^ | .026 | 13.03 | 3.35×10^-4^ | .023 | E | 6.76 | .010 | .012 | E |
| 202 | Fronto-parietal Task Control | [-3,26,44] | 6.44 | .002 | .023 | 12.89 | 3.60×10^-4^ | .023 | W | 11.96 | 5.84×10^-4^ | .021 | W |
| 206 | Salience | [31,33,26] | 14.70 | 5.99×10^-7^ | .050 | 29.43 | 8.66×10^-8^ | .050 | E | 19.28 | 1.35×10^-5^ | .033 | E |
| 207 | Salience | [48,22,10] | 11.16 | 1.78×10^-5^ | .039 | 11.43 | 7.75×10^-4^ | .020 | E | 21.29 | 4.91×10^-6^ | .037 | E |
| 217 | Salience | [10,22,27] | 6.14 | .002 | .022 | 12.05 | 5.57×10^-4^ | .021 | E | 9.39 | .002 | .017 | E |
| 222 | Subcortical | [6, 24,0] | 9.74 | 6.98×10^-5^ | .034 | 17.03 | 4.24×10^-5^ | .030 | W | 18.26 | 2.27×10^-5^ | .032 | W |
| 226 | Subcortical | [-5, 28, 4] | 16.85 | 7.83×10^-8^ | .057 | 27.22 | 2.56×10^-7^ | .047 | W | 33.05 | 1.48×10^-8^ | .056 | W |
| 227 | Subcortical | [-22,7, 5] | 6.50 | .002 | .023 | 11.86 | 6.17×10^-4^ | .021 | E | 12.88 | 3.62×10^-4^ | .023 | E |
| 232 | Subcortical | [-31, 11,0] | 7.77 | 4.72×10^-4^ | .027 | 15.50 | 9.31×10^-5^ | .027 | E | 13.76 | 2.29×10^-4^ | .024 | E |
| 234 | Subcortical | [9, 4,6] | 5.02 | .007 | .018 | 9.97 | .002 | .018 | E | 8.71 | .003 | .015 | E |
| 243 | Cerebellar | [-16, 65, 20] | 8.68 | 1.95×10^-4^ | .030 | 14.03 | 1.99×10^-4^ | .025 | E | 8.19 | .004 | .014 | E |
| 244 | Cerebellar | [-32, 55, 25] | 6.04 | .003 | .021 | 11.85 | 6.19×10^-4^ | .021 | E | 8.44 | .004 | .015 | E |
| 245 | Cerebellar | [22, 58, 23] | 8.57 | 2.16×10^-4^ | .030 | 17.05 | 4.20×10^-5^ | .030 | E | 11.83 | 6.26×10^-4^ | .021 | E |
| 257 | Dorsal attention | [46, 59,4] | 6.77 | .001 | .024 | 12.87 | 3.64×10^-4^ | .023 | E | 13.37 | 2.80×10^-4^ | .023 | E |
| 264 | Dorsal attention | [29, 5,54] | 4.73 | .009 | .017 | 9.40 | .002 | .017 | E | 7.10 | .008 | .013 | E |

*Note*. W represents a significantly higher value among Westerners; E represents a significantly higher value among East Asians (i.e., Chinese individuals).

**Table S4.** *Partial Correlation Between Network Metrics and the Analysis-Holism Scale (AHS) Based on Data that Regressed out the Global Signals*

| Nodes | | Degree | | Global efficiency | |
| --- | --- | --- | --- | --- | --- |
|  |  | *r* | *p* | *r* | *p* |
| Right dorsal superior frontal gyrus | R.SFGdor | .03 | .59 | -.03 | .61 |
| Left middle frontal gyrus | L.MFG | -.04 | .51 | -.06 | .35 |
| Right middle frontal gyrus | R.MFG | -.11 | .07 | -.15 | .02 |
| Right inferior frontal gyrus (triangular part) | R.IFGtriang | -.03 | .65 | -.04 | .51 |
| Left rolandic operculum | L.ROL | .06 | .36 | .07 | .27 |
| Right rolandic operculum | R.ROL | .06 | .35 | .04 | .56 |
| Right supplementary motor area | R.SMA | .00 | .96 | .00 | .95 |
| Left olfactory cortex | L.OLF | .08 | .20 | .07 | .27 |
| Right olfactory cortex | R.OLF | .04 | .52 | .06 | .37 |
| Left superior frontal gyrus, medial orbital | L.ORBsupmed | -.25* | 3.92×10^-5^ | -.20* | 1.59×10^-3*^ |
| Right superior frontal gyrus, medial orbital | R.ORBsupmed | -.20* | 1.37×10^-3^ | -.15 | .02 |
| Right gyrus rectus | R.REC | -.14 | .02 | -.10 | .12 |
| Left hippocampus | L.HIP | .16* | 8.79×10^-3^ | .15 | .01 |
| Left amygdala | L.AMYG | .22* | 4.68×10^-4^ | .20* | 1.07×10^-3^ |
| Right amygdala | R.AMYG | .14 | .02 | .14 | .02 |
| Left cuneus | L.CUN | -.14 | .02 | -.15 | .02 |
| Right cuneus | R.CUN | -.01 | .82 | -.05 | .39 |
| Left superior occipital gyrus | L.SOG | .01 | .91 | -.08 | .21 |
| Right superior occipital gyrus | R.SOG | .10 | .12 | -.03 | .68 |
| Left middle occipital gyrus | L.MOG | -.05 | .41 | -.10 | .11 |
| Right middle occipital gyrus | R.MOG | .04 | .52 | -.05 | .46 |
| Left superior parietal gyrus | L.SPG | .00 | .97 | .01 | .92 |
| Right angular gyrus | R.ANG | -.10 | .11 | -.11 | .07 |
| Left precuneus | L.PCUN | -.18* | 3.58×10^-3^ | -.11 | .08 |
| Right precuneus | R.PCUN | -.16* | .01 | -.09 | .13 |
| Left putamen | L.PUT | .21* | 9.14×10^-4^ | .23* | 1.81×10^-4^ |
| Right putamen | R.PUT | .19* | 1.99×10^-3^ | .21* | 6.76×10^-4^ |
| Left superior temporal gyrus | L.STG | .20* | 1.17×10^-3^ | .19* | 2.50×10^-3^ |
| Left superior temporal pole | L.TPOsup | .07 | .29 | .07 | .25 |
| Right superior temporal pole | R.TPOsup | .06 | .35 | .06 | .32 |
| Left middle temporal gyrus | L.MTG | .06 | .36 | .07 | .29 |
| Right middle temporal gyrus | R.MTG | -.05 | .41 | -.04 | .53 |

**p* < .05, FDR corrected.

**Table S5.** *Partial Correlation Between Network Metrics Calculated Based on an Absolute Network and the Analysis-Holism Scale (AHS)*

| Nodes | | Degree | | Global | |
| --- | --- | --- | --- | --- | --- |
|  |  | *r* | *p* | *r* | *p* |
| Left precental gyrus | L.PreCG | -.03 | .59 | -.04 | .56 |
| Right precental gyrus | R.PreCG | .07 | .25 | .05 | .44 |
| Left dorsal superior frontal gyrus | L.SFGdor | -.19* | 2.49×10^-3^ | -.19* | 1.87×10^-3^ |
| Left olfactory cortex | L.OLF | .00 | .97 | .01 | .82 |
| Left medial prefrontal cortex | L.mPFC | -.07 | .24 | -.08 | .18 |
| Right medial prefrontal cortex | R.mPFC | -.15 | .02 | -.13 | .04 |
| Right hippocampus | R.HIP | .11 | .09 | .06 | .36 |
| Right parahippocampal gyrus | R.PHG | -.05 | .46 | -.06 | .35 |
| Left amygdala | L.AMYG | .16* | 8.89×10^-3^ | .15 | .01 |
| Right amygdala | R.AMYG | .17* | 5.94×10^-3^ | .17* | 5.21×10^-3^ |
| Left precuneus | L.PCUN | -.14 | .02 | -.14 | .03 |
| Right precuneus | R.PCUN | -.15 | .02 | -.12 | .05 |
| Left putamen | L.PUT | .20* | 1.30×10^-3^ | .17* | 7.29×10^-3^ |
| Right putamen | R.PUT | .13 | .04 | .10 | .09 |
| Left pallidum | L.PAL | .13 | .04 | .11 | .09 |
| Right pallidum | R.PAL | .00 | .97 | .03 | .66 |
| Right superior temporal pole | R.TPOsup | .11 | .08 | .10 | .11 |
| Left middle temporal pole | L.TPOmid | .04 | .48 | .09 | .15 |

**p* < .05, FDR corrected.

**Table S6.** *Partial Correlation Between Network Metrics and the Analysis-Holism Scale (AHS) Based on Data Using the Power 264 Template*

|  |  | Degree | | Efficiency | |
| --- | --- | --- | --- | --- | --- |
| Node | Suggested network | *r* | *p* | *r* | *p* |
| 10 | Uncertain | .26* | 2.05×10^-5^ | .30* | 1.00×10^-6^ |
| 11 | Uncertain | .08 | .20 | .08 | .20 |
| 24 | Sensory/somatomotor Hand | .11 | .09 | .10 | .11 |
| 27 | Sensory/somatomotor Hand | -.02 | .77 | .02 | .71 |
| 30 | Sensory/somatomotor Hand | .08 | .20 | .07 | .25 |
| 31 | Sensory/somatomotor Hand | .03 | .59 | .02 | .71 |
| 39 | Sensory/somatomotor Hand | -.10 | .10 | -.11 | .08 |
| 40 | Sensory/somatomotor Hand | -.17* | 4.88×10^-3^ | -.20* | 1.45×10^-3^ |
| 46 | Sensory/somatomotor Mouth | -.05 | .45 | .02 | .70 |
| 49 | Cingulo-opercular Task Control | .01 | .82 | .00 | .98 |
| 61 | Auditory | -.19* | 2.47×10^-3^ | -.21* | 6.71×10^-4^ |
| 63 | Auditory | .02 | .80 | .03 | .68 |
| 64 | Auditory | -.05 | .47 | -.06 | .36 |
| 66 | Auditory | .07 | .26 | .07 | .28 |
| 67 | Auditory | .02 | .81 | -.02 | .75 |
| 73 | Auditory | .06 | .36 | .05 | .40 |
| 77 | Default mode | -.16* | 9.49×10^-3^ | -.15 | .01 |
| 82 | Default mode | .03 | .63 | .04 | .51 |
| 88 | Default mode | -.21* | 8.35×10^-4^ | -.19* | 2.78×10^-3^ |
| 89 | Default mode | -.18* | 4.14×10^-3^ | -.14 | .02 |
| 90 | Default mode | -.20* | 1.11×10^-3^ | -.20* | 1.12×10^-3^ |
| 91 | Default mode | -.10 | .10 | -.08 | .17 |
| 93 | Default mode | -.21* | 9.13×10^-4^ | -.18* | 4.21×10^-3^ |
| 95 | Default mode | -.24* | 8.18×10^-5^ | -.23* | 1.49×10^-4^ |
| 103 | Default mode | -.06 | .35 | -.07 | .26 |
| 106 | Default mode | -.11 | .09 | -.09 | .16 |
| 107 | Default mode | -.13 | .04 | -.11 | .07 |
| 109 | Default mode | -.29* | 3.07×10^-6^ | -.25* | 4.08×10^-5^ |
| 123 | Default mode | .07 | .29 | .10 | .09 |
| 124 | Default mode | -.17* | 7.76×10^-3^ | -.19* | 2.76×10^-3^ |
| 126 | Default mode | -.19* | 2.56×10^-3^ | -.19* | 1.65×10^-3^ |
| 144 | Visual | .02 | .78 | .00 | .94 |
| 147 | Visual | .03 | .68 | .00 | .98 |
| 160 | Visual | -.04 | .57 | -.06 | .38 |
| 163 | Visual | -.13 | .03 | -.14 | .03 |
| 184 | Uncertain | .03 | .60 | .00 | .98 |
| 185 | Uncertain | -.02 | .78 | .01 | .85 |
| 196 | Fronto-parietal Task Control | -.02 | .73 | -.02 | .76 |
| 202 | Fronto-parietal Task Control | -.04 | .58 | -.04 | .53 |
| 206 | Salience | .10 | .13 | .14 | .02 |
| 207 | Salience | -.01 | .84 | .02 | .79 |
| 217 | Salience | .08 | .20 | .07 | .26 |
| 222 | Subcortical | .03 | .59 | .03 | .58 |
| 226 | Subcortical | .07 | .29 | .09 | .15 |
| 227 | Subcortical | .08 | .19 | .05 | .46 |
| 232 | Subcortical | .15 | .02 | .12 | .06 |
| 234 | Subcortical | -.07 | .25 | -.01 | .82 |
| 243 | Cerebellar | .14 | .03 | .15 | .01 |
| 244 | Cerebellar | .04 | .55 | .07 | .29 |
| 245 | Cerebellar | .16* | .01 | .18* | 4.43×10^-3^ |
| 257 | Dorsal attention | -.01 | .84 | -.02 | .81 |
| 264 | Dorsal attention | .08 | .20 | .11 | .08 |

**p* < .05, FDR corrected.

**Table S7.** *Partial Correlation Analyses Between the Functional Connectivity of the Left Dorsal Superior Frontal Gyrus* *and the Analysis-Holism Scale (AHS)*

|  | *r* | *p* |
| --- | --- | --- |
| R.PreCG | -.15* | .01 |
| R.OLF | -.12 | .04 |
| L.ORBsupmed | -.16* | 6.45×10^-3^ |
| R.ORBsupmed | -.22* | 3.40×10^-4^ |
| L.REC | -.17* | 4.84×10^-3^ |
| R.REC | -.17* | 3.30×10^-3^ |
| R.ACG | -.13 | .04 |
| L.DACC | -.23* | 1.73×10^-4^ |
| R.DACC | -.16* | 8.76×10^-3^ |
| R.HIP | -.15* | .01 |
| R.PHG | -.19* | 9.13×10^-4^ |
| L.CAL | -.19* | 9.90×10^-4^ |
| R.CAL | -.21* | 2.44×10^-4^ |
| L.CUN | -.24* | 3.90×10^-5^ |
| R.CUN | -.22* | 1.67×10^-4^ |
| L.LING | -.2* | 7.68×10^-4^ |
| R.LING | -.22* | 1.58×10^-4^ |
| L.SOG | -.20* | 7.16×10^-4^ |
| R.SOG | -.16* | 5.57×10^-3^ |
| R.MOG | -.15* | .01 |
| R.IOG | -.12 | .05 |
| L.FFG | -.21* | 2.89×10^-4^ |
| R.FFG | -.21* | 3.35×10^-4^ |
| L.SPG | -.15 | .02 |
| R.ANG | -.16* | .01 |
| L.PCUN | -.18* | 1.81×10^-3^ |
| R.PCUN | -.16* | 7.47×10^-3^ |
| R.PCL | -.13 | .03 |
| L.CAU | -.15* | .01 |
| R.CAU | -.14 | .02 |
| R.PUT | -.17* | 6.72×10^-3^ |
| R.STG | -.13 | .03 |
| L.MTG | -.14 | .02 |
| R.MTG | -.15* | .01 |
| R.TPO | -.17* | 4.72×10^-3^ |

*Note.* Please see Table S11 for the full names of the abbreviations.

*p < 0.05, FDR corrected.

**Table S8.** *Partial Correlation Analyses Between the Functional Connectivity of the Right Medial Prefrontal Cortex and the Analysis-Holism Scale (AHS)*

|  | *r* | *p* |
| --- | --- | --- |
| R.PreCG | -.14 | .02 |
| L.OLF | -.13 | .02 |
| L.ORBsupmed | -.24* | 6.00×10^-5^ |
| R.ORBsupmed | -.19* | 1.02×10^-3^ |
| L.REC | -.22* | 1.30×10^-4^ |
| R.REC | -.19* | 1.12×10^-3^ |
| L.ACG | -.18* | 3.57×10^-3^ |
| R.ACG | -.16* | .01 |
| L.DACC | -.22* | 1.70×10^-4^ |
| R.DACC | -.13 | .03 |
| L.PCG | -.17* | 4.28×10^-3^ |
| L.HIP | -.14 | .02 |
| R.HIP | -.15* | .01 |
| L.PHG | -.12 | .05 |
| R.PHG | -.18* | 1.85×10^-3^ |
| L.AMYG | -.16* | 9.44×10^-3^ |
| L.CAL | -.19* | 1.00×10^-3^ |
| R.CAL | -.21* | 4.10×10^-4^ |
| L.CUN | -.23* | 1.30×10^-4^ |
| R.CUN | -.21* | 5.20×10^-4^ |
| L.LING | -.18* | 2.48×10^-3^ |
| R.LING | -.17* | 3.76×10^-3^ |
| L.SOG | -.18* | 3.22×10^-3^ |
| R.SOG | -.17* | 5.99×10^-3^ |
| L.FFG | -.18* | 2.74×10^-3^ |
| R.FFG | -.13 | .04 |
| L.PCUN | -.18* | 2.74×10^-3^ |
| L.PCL | -.12 | .04 |
| L.CAU | -.18* | 3.55×10^-3^ |
| L.PUT | -.17* | 7.94×10^-3^ |
| R.PUT | -.17* | 8.07×10^-3^ |
| R.HES | -.12 | .05 |
| R.STG | -.14 | .02 |
| L.MTG | -.16* | 6.21×10^-3^ |
| R.MTG | -.12 | .04 |
| R.TPO | -.15* | .01 |

*Note.* Please see Table S11 for the full names of the abbreviations.

*p < 0.05, FDR corrected.

**Table S9.** *Partial Correlation Analyses Between the Functional Connectivity of the Left Precuneus and the Analysis-Holism Scale (AHS)*

|  | *r* | *p* |
| --- | --- | --- |
| L.SFGdor | -.18* | 1.81×10^-3^ |
| R.SFGdor | -.15 | .01 |
| L.OLF | -.14 | .02 |
| L. mPFC | -.15 | .01 |
| R. mPFC | -.18* | 2.74×10^-3^ |
| L.ORBsupmed | -.19* | 1.41×10^-3^ |
| R.ORBsupmed | -.21* | 3.53×10^-4^ |
| L.REC | -.13 | .03 |
| L.ACG | -.12 | .05 |
| L.PCG | -.21* | 2.35×10^-4^ |
| R.PCG | -.20* | 3.88×10^-4^ |
| L.CAL | -.15 | 9.38×10^-3^ |
| R.CAL | -.13 | .03 |
| L.CUN | -.14 | .02 |
| L.LING | -.14 | .02 |
| R.LING | -.13 | .03 |
| L.SOG | -.16 | 7.85×10^-3^ |
| R.SOG | -.13 | .03 |
| L.MOG | -.21* | 2.57×10^-4^ |
| R.MOG | -.16* | 5.01×10^-3^ |
| L.FFG | -.16* | 5.44×10^-3^ |
| R.FFG | -.13 | .03 |
| L.SPG | -.15 | .01 |
| L.ANG | -.15 | 9.77×10^-3^ |
| R.ANG | -.15 | .01 |
| R.PCUN | -.17* | 6.64×10^-3^ |
| L.CAU | -.20* | 7.35×10^-4^ |
| R.CAU | -.17* | 5.96×10^-3^ |
| R.TPO | -.14 | .01 |
| L.ITG | -.12 | .05 |

*Note.* Please see Table S11 for full names of the abbreviations.

*p < 0.05, FDR corrected.

**Table S10.** *Partial Correlation Analyses Between the Functional Connectivity of the Right Precuneus and the Analysis-Holism Scale (AHS)*

|  | *r* | *p* |
| --- | --- | --- |
| L.SFGdor | -.16 | 7.47×10^-3^ |
| L.OLF | -.17 | 5.72×10^-3^ |
| L.ORBsupmed | -.14 | .02 |
| R.ORBsupmed | -.14 | .02 |
| L.PCG | -.13 | .02 |
| L.SOG | -.13 | .03 |
| L.MOG | -.21* | 5.27×10^-4^ |
| R.MOG | -.13 | .03 |
| L.FFG | -.13 | .03 |
| L.SPG | -.19* | 1.53×10^-3^ |
| R.SPG | -.14 | .02 |
| L.PCUN | -.17 | 6.64×10^-3^ |
| R.PCL | -.15 | .01 |
| L.CAU | -.20* | 8.33×10^-4^ |
| R.CAU | -.14 | .02 |

*Note*. Please see Table S11 for the full names of the abbreviations.

**p* < 0.05, FDR corrected.

**Table S11.** *Abbreviations and Functional Classification of all Nodes in Automated Anatomical Labeling Atlas (AAL-90)*

| Nodes | Abbreviation | Functional classification |
| --- | --- | --- |
| Left precental gyrus | L.PreCG | Primary |
| Right precental gyrus | R.PreCG | Primary |
| Left dorsal superior frontal gyrus | L.SFGdor | Association |
| Right dorsal superior frontal gyrus | R.SFGdor | Association |
| Left superior frontal gyrus, orbital part | L.ORBsup | ParaLimbic |
| Right superior frontal gyrus, orbital part | R.ORBsup | ParaLimbic |
| Left middle frontal gyrus | L.MFG | Association |
| Right middle frontal gyrus | R.MFG | Association |
| Left middle frontal gyrus (orbital part) | L.ORBmid | ParaLimbic |
| Right middle frontal gyrus (orbital part) | R.ORBmid | ParaLimbic |
| Left inferior frontal gyrus (opercular part) | L.IFGoperc | Association |
| Right inferior frontal gyrus (opercular part) | R.IFGoperc | Association |
| Left inferior frontal gyrus (triangular part) | L.IFGtriang | Association |
| Right inferior frontal gyrus (triangular part) | R.IFGtriang | Association |
| Left inferior frontal gyrus (orbital part) | L.ORBinf | ParaLimbic |
| Right inferior frontal gyrus (orbital part) | R.ORBinf | ParaLimbic |
| Left rolandic operculum | L.ROL | Association |
| Right rolandic operculum | R.ROL | Association |
| Left supplementary motor area | L.SMA | Association |
| Right supplementary motor area | R.SMA | Association |
| Left olfactory cortex | L.OLF | Limbic |
| Right olfactory cortex | R.OLF | Limbic |
| Left medial prefrontal cortex | L.mPFC | Association |
| Right medial prefrontal cortex | R.mPFC | Association |
| Left superior frontal gyrus, medial orbital | L.ORBsupmed | ParaLimbic |
| Right superior frontal gyrus, medial orbital | R.ORBsupmed | ParaLimbic |
| Left gyrus rectus | L.REC | ParaLimbic |
| Right gyrus rectus | R.REC | ParaLimbic |
| Left insula | L.INS | ParaLimbic |
| Right insula | R.INS | ParaLimbic |
| Left anterior cingulate and paracingulate gyri | L.ACG | ParaLimbic |
| Right anterior cingulate and paracingulate gyri | R.ACG | ParaLimbic |
| Left dorsal anterior cingulate cortex | L.DACC | ParaLimbic |
| Right dorsal anterior cingulate cortex | R.DACC | ParaLimbic |
| Left posterior cingulate gyrus | L.PCG | ParaLimbic |
| Right posterior cingulate gyrus | R.PCG | ParaLimbic |
| Left hippocampus | L.HIP | Limbic |
| Right hippocampus | R.HIP | Limbic |
| Left parahippocampal gyrus | L.PHG | ParaLimbic |
| Right parahippocampal gyrus | R.PHG | ParaLimbic |
| Left amygdala | L.AMYG | Subcortical |
| Right amygdala | R.AMYG | Subcortical |
| Left calcarine fissure and surrounding cortex | L.CAL | Primary |
| Right calcarine fissure and surrounding cortex | R.CAL | Primary |
| Left cuneus | L.CUN | Association |
| Right cuneus | R.CUN | Association |
| Left lingual gyrus | L.LING | Association |
| Right lingual gyrus | R.LING | Association |
| Left superior occipital gyrus | L.SOG | Association |
| Right superior occipital gyrus | R.SOG | Association |
| Left middle occipital gyrus | L.MOG | Association |
| Right middle occipital gyrus | R.MOG | Association |
| Left inferior occipital gyrus | L.IOG | Association |
| Right inferior occipital gyrus | R.IOG | Association |
| Left fusiform gyrus | L.FFG | Association |
| Right fusiform gyrus | R.FFG | Association |
| Left postcentral gyrus | L.PoCG | Primary |
| Right postcentral gyrus | R.PoCG | Primary |
| Left superior parietal gyrus | L.SPG | Association |
| Right superior parietal gyrus | R.SPG | Association |
| Left inferior parietal | L.IPL | Association |
| Right inferior parietal | R.IPL | Association |
| Left supramarginal gyrus | L.SMG | Association |
| Right supramarginal gyrus | R.SMG | Association |
| Left angular gyrus | L.ANG | Association |
| Right angular gyrus | R.ANG | Association |
| Left precuneus | L.PCUN | Association |
| Right precuneus | R.PCUN | Association |
| Left paracentral lobule | L.PCL | Association |
| Right paracentral lobule | R.PCL | Association |
| Left caudate | L.CAU | Subcortical |
| Right caudate | R.CAU | Subcortical |
| Left putamen | L.PUT | Subcortical |
| Right putamen | R.PUT | Subcortical |
| Left pallidum | L.PAL | Subcortical |
| Right pallidum | R.PAL | Subcortical |
| Left thalamus | L.THA | Subcortical |
| Right thalamus | R.THA | Subcortical |
| Left heschl gyrus | L.HES | Primary |
| Right heschl gyrus | R.HES | Primary |
| Left superior temporal gyrus | L.STG | Association |
| Right superior temporal gyrus | R.STG | Association |
| Left superior temporal pole | L.TPOsup | ParaLimbic |
| Right superior temporal pole | R.TPOsup | ParaLimbic |
| Left middle temporal gyrus | L.MTG | Association |
| Right middle temporal gyrus | R.MTG | Association |
| Left middle temporal pole | L.TPOmid | ParaLimbic |
| Right middle temporal pole | R.TPOmid | ParaLimbic |
| Left inferior temporal gyrus | L.ITG | Association |
| Right inferior temporal gyrus | R.ITG | Association |

*Note.* This table is adopted from the GRETNA toolbox (http://www.nitrc.org/projects/gretna/, J. Wang et al., 2015).
